# Direct and indirect genetic effects on early neurodevelopmental traits

**DOI:** 10.1111/jcpp.14122

**Published:** 2025-01-30

**Authors:** Laura Hegemann, Espen Eilertsen, Johanne Hagen Pettersen, Elizabeth C. Corfield, Rosa Cheesman, Leonard Frach, Ludvig Daae Bjørndal, Helga Ask, Beate St Pourcain, Alexandra Havdahl, Laurie J. Hannigan

**Affiliations:** ^1^ Department of Psychology University of Oslo Oslo Norway; ^2^ Research Department Lovisenberg Diaconal Hospital Oslo Norway; ^3^ PsychGen Center for Genetic Epidemiology and Mental Health Norwegian Institute of Public Health Oslo Norway; ^4^ Department of Psychology, PROMENTA Research Center University of Oslo Oslo Norway; ^5^ Department of Child Health and Development Norwegian Institute of Public Health Oslo Norway; ^6^ MRC Integrative Epidemiology Unit (IEU) University of Bristol Bristol UK; ^7^ Division of Psychology and Language Sciences, Department of Clinical, Educational and Health Psychology University College London London UK; ^8^ Language and Genetics Department Max Planck Institute for Psycholinguistics Nijmegen The Netherlands; ^9^ Donders Institute for Brain, Cognition and Behaviour Radboud University Nijmegen The Netherlands

**Keywords:** Autism, ADHD, MoBa, indirect genetic effects, genetic nurture, neurodevelopmental traits

## Abstract

**Background:**

Neurodevelopmental conditions are highly heritable. Recent studies have shown that genomic heritability estimates can be confounded by genetic effects mediated via the environment (indirect genetic effects). However, the relative importance of direct versus indirect genetic effects on early variability in traits related to neurodevelopmental conditions is unknown.

**Methods:**

The sample included up to 24,692 parent‐offspring trios from the Norwegian MoBa cohort. We use Trio‐GCTA to estimate latent direct and indirect genetic effects on mother‐reported neurodevelopmental traits at age of 3 years (restricted and repetitive behaviors and interests, inattention, hyperactivity, language, social, and motor development). Further, we investigate to what extent direct and indirect effects are attributable to common genetic variants associated with autism, ADHD, developmental dyslexia, educational attainment, and cognitive ability using polygenic scores (PGS) in regression modeling.

**Results:**

We find evidence for contributions of direct and indirect latent common genetic effects to inattention (direct: explaining 4.8% of variance, indirect: 6.7%) hyperactivity (direct: 1.3%, indirect: 9.6%), and restricted and repetitive behaviors (direct: 0.8%, indirect: 7.3%). Direct effects best explained variation in social and communication, language, and motor development (5.1%–5.7%). Direct genetic effects on inattention were captured by PGS for ADHD, educational attainment, and cognitive ability, whereas direct genetic effects on language development were captured by cognitive ability, educational attainment, and autism PGS. Indirect genetic effects on neurodevelopmental traits were primarily captured by educational attainment and/or cognitive ability PGS.

**Conclusions:**

Results were consistent with differential contributions to neurodevelopmental traits in early childhood from direct and indirect genetic effects. Indirect effects were particularly important for hyperactivity and restricted and repetitive behaviors and interests and may be linked to genetic variation associated with cognition and educational attainment. Our findings illustrate the importance of within‐family methods for disentangling genetic processes that influence early neurodevelopmental traits, even when identifiable associations are small.

## Introduction

Neurodevelopmental conditions, such as autism and attention‐deficit hyperactivity disorder (ADHD), are highly heritable (Gidziela et al., 2023) and have the earliest age‐of‐onset of neuropsychiatric conditions (Solmi et al., 2022). Numerous early indicators – observable prior to the ages at which these conditions are typically diagnosed – have been robustly associated with later neurodevelopmental diagnoses. These include early behavioral traits (Micai et al., 2020), infant developmental milestones (Johnson, Gliga, Jones, & Charman, 2015), and even intrauterine (Levine et al., 2015) and gestational (Ask et al., 2018) factors.

In many cases, early indicators of neurodevelopmental conditions have been shown to correlate with genetic liabilities that underpin the conditions themselves (Askeland et al., 2022; Hannigan et al., 2021; Riglin et al., 2022; Serdarevic et al., 2020). Most parsimoniously, these findings indicate that genetic liability to neurodevelopmental disorders manifests behaviorally in early life. However, it is important to consider alternative explanations of these findings. The sharing of genetic material between parents and children means that even the earliest environments to which infants are exposed are correlated with their genotypes (Havdahl et al., 2022; Leppert et al., 2019). Additionally, due to the correlation between parental and child genotypes, biases arising from assortative mating, selection processes, population stratification, and rater effects can impact observed associations between a child's genotype and potential early indicators (McAdams, Cheesman, & Ahmadzadeh, 2023; Young, Benonisdottir, Przeworski, & Kong, 2019). This has implications not only for any assumptions we might make about the role of early *environments* for neurodevelopmental conditions, but also for interpreting their heritability – and any early correlates of their genetic liabilities.

Effects of an individual's own genotype on their own behavior, or *direct genetic effects*, are commonly assumed to be the main contributor to heritability estimates. However, another key source of heritable variation is *indirect genetic effects* – where variation in a trait in one individual is caused by the genotype of another individual (Bijma, 2014; Wolf, Brodie III, Cheverud, Moore, & Wade, 1998). In the case of indirect effects of parent's genotypes on their child's phenotypes, these are sometimes referred to as ‘genetic nurture’ effects (Kong et al., 2018). Of all the factors outside of direct effects that can influence estimates of the heritability (Yang, Zeng, Goddard, Wray, & Visscher, 2017), indirect genetic effects are particularly interesting to isolate, because they represent – at least theoretically – environmental processes detectable via reliably measured genotypic information.

Within‐family genetic analyses can be used to disentangle direct and indirect genetic effects on neurodevelopmental traits and conditions (McAdams et al., 2023). Genomic estimates of the heritability of neurodevelopment‐linked traits such as educational attainment and cognition are reduced by as much as 50% when estimated in within‐family models (Howe et al., 2022), suggesting that these traits may be particularly influenced by indirect effects and/or mechanisms such as assortative mating and population stratification. Likewise, the prediction of ADHD traits by educational attainment polygenic liability at age 12 is substantially (>50%) reduced in a within‐family paradigm (Selzam et al., 2019). Nonetheless, *direct* genetic effects remain influential – for example, direct genetic effects on ADHD diagnoses are captured in polygenic scores (PGS) for ADHD, obsessive‐compulsive disorder, and cognitive ability (Martin et al., 2023). Among 8‐year‐old children in the Norwegian Mother, Father, and Child Cohort study (MoBa), two studies find evidence consistent with a role for both direct and indirect genetic effects – which explain, respectively, 10%–24% and 8%–16% of the variance in ADHD traits (Eilertsen et al., 2022; Jami et al., 2023). An alternative approach in the same dataset finds that only maternal genetic liability for neuroticism is a robust source of indirect genetic effects on 8‐year‐olds ADHD traits; whereas children's genetic liability to ADHD, smoking, educational attainment, and cognitive ability are all potential sources of direct genetic effects (Pingault et al., 2023).

Little is known about the relative importance of direct and indirect genetic effects on early life neurodevelopment. Here, we address this knowledge gap using measures of early neurodevelopmental traits obtained from mothers of 3‐year‐old children in the MoBa cohort. Investigating dimensional traits in the general population, as opposed to clinical diagnoses, can not only offer more statistical power but also give nuanced insight into how genetic variation of these conditions influences early behavioral traits (e.g., social communication or inattention) prior to the typical age of diagnosis. Additionally, studies have shown strong genetic links (Martin, Taylor, & Lichtenstein, 2018) and similar variances explained by shared and nonshared environmental influences   between continuous traits and clinical diagnoses (Gidziela et al., 2023). Therefore, in this study, using relevant dimensional outcomes and two complementary within‐family approaches, we address two main questions. First, with the Trio‐GCTA method: to what extent are genetic effects on early life neurodevelopmental traits direct versus indirect? Second, with the trio‐PGS method: can these effects be attributed to established polygenic liabilities for neurodevelopmental conditions and related traits?

## Methods

### Measures and sample

#### Sample

MoBa is a population‐based pregnancy cohort study conducted by the Norwegian Institute of Public Health (Magnus et al., 2006, 2016). Participants were recruited from all over Norway from 1999 to 2008. The women consented to participation in 41% of the pregnancies. Blood samples were obtained from both parents during pregnancy and from mothers and children (umbilical cord) at birth (Paltiel et al., 2014). The cohort includes ~114,500 children, 95,200 mothers, and 75,200 fathers. The current study is based on version 12 of the quality‐assured data files released for research in January 2019. The establishment of MoBa and initial data collection was based on a license from the Norwegian Data Protection Agency and approval from The Regional Committees for Medical and Health Research Ethics. The MoBa cohort is currently regulated by the Norwegian Health Registry Act. The current study was approved by The Regional Committees for Medical and Health Research Ethics (2016/1702). Child's sex was ascertained from the Medical Birth Registry (MBRN) which is a national health registry containing information about all births in Norway.

The present study was conducted on a subset of the cohort who were complete genotyped family trios and had information available from the 3‐year questionnaire (*N*_trio_ = 24,692). For each measure, individuals were removed if they had more than 50% of the items in a measure missing or were older than 4 years when the questionnaire was returned. The sample for the Trio‐GCTA analyses was further restricted to unrelated trios (*N*_trio_ = 16,565) to limit potential confounding due to closely related individuals (Yang et al., 2017). This was done by removing any individuals that had genetic relatedness above 0.1, notwithstanding parent/child relationships, using the ‘bottom up’ algorithm in OpenMendel (Zhou et al., 2020). This resulted in sample sizes of 24,550–24,621 for the trio‐PGS and 16,518–16,565 for Trio‐ GCTA analyses, depending on the outcome. For more information on genotyping of the MoBa sample and for the family‐based quality control pipeline used to prepare these data for analysis, see Corfield et al. (2022).

#### Measures for neurodevelopmental traits

The current (as opposed to lifetime) version of the *Social Communication Questionnaire* (SCQ) was used as a measure of social communication traits as well as repetitive and restrictive behaviors and interests (RRBI). The SCQ was split into subscales of social communication and RRBI based on DSM‐5 diagnostic criteria for autism. As a post hoc analysis, a SCQ total score was also used. Items from the *Child Behavior Checklist* (CBCL) were used to measure inattention and hyperactivity. Fine and gross motor and receptive and expressive language development were measured using items from the *Ages and Stages Questionnaire* (ASQ). Measures and included items were selected based on relevance and availability in the age 3 MoBa questionnaire. The exact items going into the scales can be found in the Appendix S2. Only overall motor and language scales (not subscales for fine/gross motor or receptive/expressive language) could be used in analyses that required continuous measures due to excessively skewed distributions. The fine motor and expressive language development subscales were treated as ordinal while receptive language and gross motor development were dichotomized and interpreted as no versus any reported difficulties in these areas.

Sum scores for each measure were calculated for individuals with <50% missingness of the items, by taking a mean of available items and multiplying by the scale length. A square root transformation was performed on the measures of social communication traits, language, and motor development due to moderate right skewness and all measures were standardized to have a mean of 0 and standard deviation of 1. Items were reverse coded when necessary, so that for all scores higher values reflected the presence of more neurodevelopmental divergence.

#### Polygenic scores

PGS were calculated using the software LDpred2 (Privé, Arbel, & Vilhjálmsson, 2020). Recommended quality control steps were followed (Privé, Arbel, & Vilhjálmsson, 2020) using an established pipeline (Allegrini, ) which included restricting variants to an extended set of HAPMAP3+ variants (1.4 million SNPs). Precomputed LD matrixes from UK Biobank were used as the reference LD panel (Privé, Albiñana, Arbel, Pasaniuc, & Vilhjálmsson, 2023). Scores were calculated using the option ‘LDPred2‐auto’. We used summary statistics from the most recent large‐scale (>10,000 cases) GWAS of neurodevelopmental conditions. These were ADHD (Demontis et al., 2023), autism (Grove et al., 2019), and dyslexia (Doust et al., 2022). The PGS for dyslexia was created using the publicly available summary statistics containing only the top 10,000 SNPs. In addition to including PGS for these neurodevelopmental conditions, PGS for educational attainment (Lee et al., 2018) and cognitive ability (Savage et al., 2018) were also calculated as they are related traits to neurodevelopment. The educational attainment PGS was constructed from summary statistics excluding 23andMe and MoBa participants. There is no direct overlap of MoBa participants and the other GWAS used. PGS were regressed on the first 20 genomic principal components (PCs), genotype, and imputation batch. PGS were also created using a clumping and threshold method as specified in the preregistration, results of the analyses using these scores are presented in the Supporting Information.

### Analyses

#### Trio‐GCTA

To estimate the latent direct and indirect common genetic effects underlying early neurodevelopmental traits, Trio‐GCTA was used (Eilertsen et al., 2021). Trio‐GCTA, an extension of earlier GCTA (Yang et al., 2010; Yang, Lee, Goddard, & Visscher, 2011) methods, was developed to disentangle direct and indirect genetic effects. In this model, the additive genetic variance effects on an outcome from all measured single‐nucleotide polymorphisms (SNP) of all members of parent‐offspring trios are estimated simultaneously. Direct and indirect genetic effects are partitioned using a linear mixed model and variance components for all three members of the trios are estimated. In the current study, the child is the focal individual in the trio (i.e., the ‘owner’ of the phenotype), so the estimated variance of the child's genetic effects was interpreted as direct genetic effects. The estimated variances of the mother and father's genetic effects were interpreted as maternal and paternal indirect effects, respectively. In a ‘full’ model, this includes genetic effects of the child ($\sigma_{c}^{2}$; direct effect), mother ($\sigma_{m}^{2}$; indirect effect), and father ($\sigma_{p}^{2}$; indirect effect). Additionally, covariances between genetic effects from the child and mother ($\sigma_{\mathrm{om}}$), child and father ($\sigma_{\mathrm{op}}$), father and mother ($\sigma_{\mathrm{mp}}$), as well as the residual variance$\;\left(\sigma_{e}^{2}\right)$, are estimated. The total variance decomposition of the phenotype is shown in Equation 1. Covariance between the father and mother was excluded in the calculation of the total variance, under the assumption that parents are unrelated and have an expected genetic relatedness of 0.

(1) $$\mathrm{Var}\left(Y\right)=\sigma_{m}^{2}+\sigma_{p}^{2}+\sigma_{c}^{2}+\sigma_{\mathrm{om}}+\sigma_{\mathrm{op}}+\sigma_{e}^{2}$$

We compared this ‘full’ model to a nested model omitting the covariances between child/parent and parents' genetic effects (‘No covariance’). This ‘no covariance’ model, in turn, was compared with a nested model with only direct effects (‘direct only’), which was subsequently compared with a ‘null’ model including no genetic effects at all (Table 1). Model fit was assessed using Akaike's Information Criteria (AIC). Additionally, Bayesian Information Criteria (BIC) was reported. We chose AIC because of its efficiency, compared with BIC, at selecting the model closest to the true model when the true model is not present in the candidate models – particularly when the true model is complex and/or contains small effects (Vrieze, 2012). Finally, a likelihood ratio test was performed comparing the nested models for each outcome providing additional insight into the statistical significance of removing parameters from the model. *p*‐values from these tests were corrected for six tests (based on six outcomes) using the false discovery method (FDR; Benjamini and Hochberg, 1995). Estimates of variance components were interpreted from the best‐fitting model, but results from all models are presented given potential discrepancies in model selection.

**Table 1:** Variance decomposition of the four models tested in the Trio‐GCTA analyses

Model	Variance decomposition
Full	$\sigma_{c}^{2}+\sigma_{p}^{2}+\sigma_{m}^{2}+\sigma_{\mathrm{om}}+\sigma_{\mathrm{op}}+\sigma_{e}^{2}$
No covariance	$\sigma_{c}^{2}+\sigma_{p}^{2}+\sigma_{m}^{2}+\sigma_{e}^{2}$
Direct only	$\sigma_{c}^{2}+\sigma_{e}^{2}$
Null	$\sigma_{e}^{2}$

The variance decomposition is based on three subsets (mothers, children, fathers) of a genomic relatedness matrix of the full sample (Eilertsen et al., 2021). The four nested models were run in Trio‐GCTA for the six continuous neurodevelopmental trait measures: attention, hyperactivity, RRBI, social communication traits, motor, and language development. All models included fixed effects for child's sex, genotyping batch, and first 10 genomic PCs.

#### Trio‐PGS regression modeling

Regression modeling was used to investigate direct and indirect effects attributable to PGS of neurodevelopmental conditions and related traits on measures of early neurodevelopment. The six continuous neurodevelopmental trait measures were used as outcomes in separate linear regression models. These measures were regressed onto the five PGS traits in independent models. In total, this resulted in 30 models (6 continuous outcomes, 5 PGS traits) including the mother, father, and child PGS as predictors in each model. Jointly considering the effects of all family members in the trios allows for disentangling direct (transmitted) genetic effects from indirect genetic effects (Pingault et al., 2023). Effect estimates from these models were interpreted as direct ($\beta_{1}$; child's PGS) and indirect effects ($\beta_{2}$ and $\beta_{3}$; mother and father's PGS) of the PGS trait on the outcome. We also ran analyses with subdomains of motor and language development as outcomes, treating gross motor and receptive language subdomains as binary, and fine motor and expressive language subdomains as ordinal.

A clustered sandwich estimator (Zeileis, 2004; Zeileis, Köll, & Graham, 2020) was used to estimate standard errors for all models, with individuals clustered on maternal ID to account for nuclear family structures in the MoBa. Sex and age at the time of questionnaires return date were included as covariates in the models, meaning that the standard model for a given outcome was as follows:

(2) $$\text{Outcome}=\begin{array}{l} \beta_{0}+\beta_{1}\mathrm{cPG}S_{\text{trait}}+\beta_{2}\mathrm{mPG}S_{\text{trait}}\\ +\;\beta_{3}\mathrm{fPG}S_{\text{trait}}+\beta_{4}\text{cSex}+\beta_{5}\text{cAgeQreturn} \end{array}$$

*p*‐values were adjusted for multiple testing across all regression analyses. These were calculated using the FDR method, correcting for 50 tests (10 outcomes × 5 PGS traits). The 10 outcomes are the 6 continuous outcomes presented in the main text to align with the Trio‐GCTA analyses and the 4 preregistered non‐continuous subdomains of motor and language presented primarily in the Supporting Information.

In addition to models looking at the effects of the PGS on neurodevelopmental traits individually (‘single trait trio‐PGS’), a model with all PGS traits included (‘multi‐trait trio‐PGS’) was also run for each of the six continuous measures. As a sensitivity analysis, these models were also run restricting the sample to only include one child per family (*N*_trio_ = 21,893 – 21,951) to account for potential confounding from siblings sharing both genetic material and (likely) home environments. For these models, the total variance and the variance explained due to direct and indirect effects by all the PGS combined were interpreted. The variance explained due to direct and indirect effects was calculating the partial *r* ^2^ estimates of the PGS for mothers and fathers (indirect effects) and children (direct effects) in these models.

#### Software

All regression analyses and preparation of phenotypic data were run in R version 4.1.2 (R Core Team, 2021). Questionnaire data were processed using the phenotools (v0.3.0) R package (Hannigan et al., 2023). Version 1.12.2 of the R package bigsnpr (Privé, Aschard, Ziyatdinov, & Blum, 2018) was used to create the PGS. Trio‐PGS regression models and estimation of clustered standard errors were conducted using the R packages stats (v4.1.2) (R Core Team, 2021), MASS (v7.3‐54) (Venables, Ripley, & Venables, 2002), sandwich (v3.01) (Zeileis, 2004, 2006; Zeileis et al., 2020), and lmtest (v0.9‐38) (Zeileis & Hothorn, 2002). Partial *r* ^2^ estimates were calculated with the R package sensemakr (v0.1.6) (Cinelli, Ferwerda, Hazlett, & Rudkin, 2024; Cinelli & Hazlett, 2019). Trio‐GCTA models were run using the VCModels (v1.0.0) (Eilertsen, 2023) in version 1.6.2 of Julia (Bezanson, Edelman, Karpinski, & Shah, 2017).

#### Preregistration and publicly available code

This study was pre‐registered. The preregistration is available at: https://doi.org/10.17605/OSF.IO/E2JQ3. Due to review suggestions and constraints on analytic possibilities resulting from the observed distributions of the data, we deviated from the preregistered analysis plan. An overview of the deviations and justifications for making them is presented in the Appendix S1.

Analytic code can be found at https://github.com/psychgen/within‐fam‐early‐neurodev‐moba.

## Results

For each of the measures of neurodevelopmental traits used in the main analyses, the maximum *N* available for analysis (i.e., with non‐missing genotypic and phenotypic data) and distributional properties are shown in Table 2. Endorsement rates of the subdomains of motor (gross and fine motor) and language (expressive and receptive) development are given in Table S1. The children were on average 3.1 years old when mothers completed the questionnaire. The sample had a 1.05:1 male‐to‐female ratio.

**Table 2:** Descriptive information on the six continuous measures of early neurodevelopmental traits

Scales	*N*	Min	Max	Mean	*SD*	Skew	Kurtosis	Tr. Skew	Tr. Kurtosis	Alpha
Social and communication (SCQ)	24,599	0	22	2.23	1.73	1.74	7.2	0.58	1.07	0.65
RRBI (SCQ)	24,558	0	12	3.77	2.49	0.63	−0.09			0.73
Inattention (CBCL)	24,556	0	6	1.47	1.27	0.81	0.33			0.62
Hyperactivity (CBCL)	24,550	0	6	1.96	1.37	0.42	−0.11			0.62
Motor (ASQ)	24,548	0	8	1.17	1.34	1.18	1.09	0.7	−0.44	0.46
Language (ASQ)	24,621	0	12	0.62	1.11	3.62	21.91	1.99	5.83	0.76

Alpha, std. Cronbach alpha; *SD*, standard deviation; Tr., transformed scale.

### Selection and selective attrition

We tested the extent to which our analytic sample differed from the overall MoBa sample in two ways: by comparing individuals with and without genotyping information on the age 3 trait measures, and by comparing individuals with and without age 3 trait measures on the five PGS. The results are presented in Tables S2 and S3. We found children with genotyping information available had significantly fewer reported neurodevelopmental difficulties in all areas, except motor development. Parents of and children with the age 3 data available had significantly lower PGS for ADHD and dyslexia (mothers and children only) and significantly higher PGS for educational attainment and cognitive ability than those missing the age 3 data.

### Trio‐GCTA

The results of the Trio‐GCTA model fitting are presented in Table 3. We find evidence for direct and – for inattention, hyperactivity, and RRBI – indirect latent genome‐wide genetic effects. A model with uncorrelated direct and indirect effects was the best‐fitting model using the AIC criterion for inattention, hyperactivity, and RRBI. For early social communication traits, language, and motor development, the simpler model including only direct effects was favored.

**Table 3:** Akaike's information criteria (AIC), Bayesian information criteria (BIC), and FDR corrected *p*‐values for the likelihood ratio test presented for the four models run for each continuous neurodevelopmental outcome in the Trio‐GCTA models

Model	−2ll	AIC	BIC	*df*	Δ*df*	Adj. *p*‐value
Inattention						
Full	46,686.23	46,770.23	47,094.16	42		
No covariance	46,687.96	**46,765.96**	47,066.75	39	−3	.914
Direct only	46,698.83	46,772.83	47,058.19	37	−2	.013
Null	46,711.79	46,783.79	47,061.44	36	−1	.002
Hyperactivity						
Full	46,894.51	46,978.51	47,302.42	42		
No covariance	46,896.07	**46,974.07**	47,274.85	39	−3	.914
Direct only	46,914.78	46,988.78	47,274.13	37	−2	<.001
Null	46,921.15	46,993.15	47,270.79	36	−1	.016
Language						
Full	45,695.36	45,779.36	46,103.38	42		
No covariance	45,696.52	45,774.52	46,075.4	39	−3	.914
Direct only	45,697.5	**45,771.5**	46,056.95	37	−2	.735
Null	45,705.78	45,777.78	46,055.51	36	−1	.008
Motor						
Full	45,484.6	45,568.6	45,892.5	42		
No covariance	45,484.73	45,562.73	45,863.49	39	−3	.989
Direct only	45,487.99	**45,561.99**	45,847.33	37	−2	.294
Null	45,494.1	45,566.1	45,843.72	36	−1	.016
RRBI						
Full	46,117.38	46,201.38	46,525.35	42		
No covariance	46,121.59	**46,199.59**	46,500.41	39	−3	.802
Direct only	46,131.58	46,205.58	46,490.97	37	−2	.013
Null	46,134.76	46,206.76	46,484.45	36	−1	.074
Social communication						
Full	46,110.86	46,194.86	46,518.89	42		
No covariance	46,114.81	46,192.81	46,493.69	39	−3	.802
Direct only	46,114.86	**46,188.86**	46,474.31	37	−2	.974
Null	46,124.09	46,196.09	46,473.84	36	−1	.007

AIC bolded for best‐fitting model. −2ll, −2 log likelihood; df, degrees of freedom.

**Figure 1:**
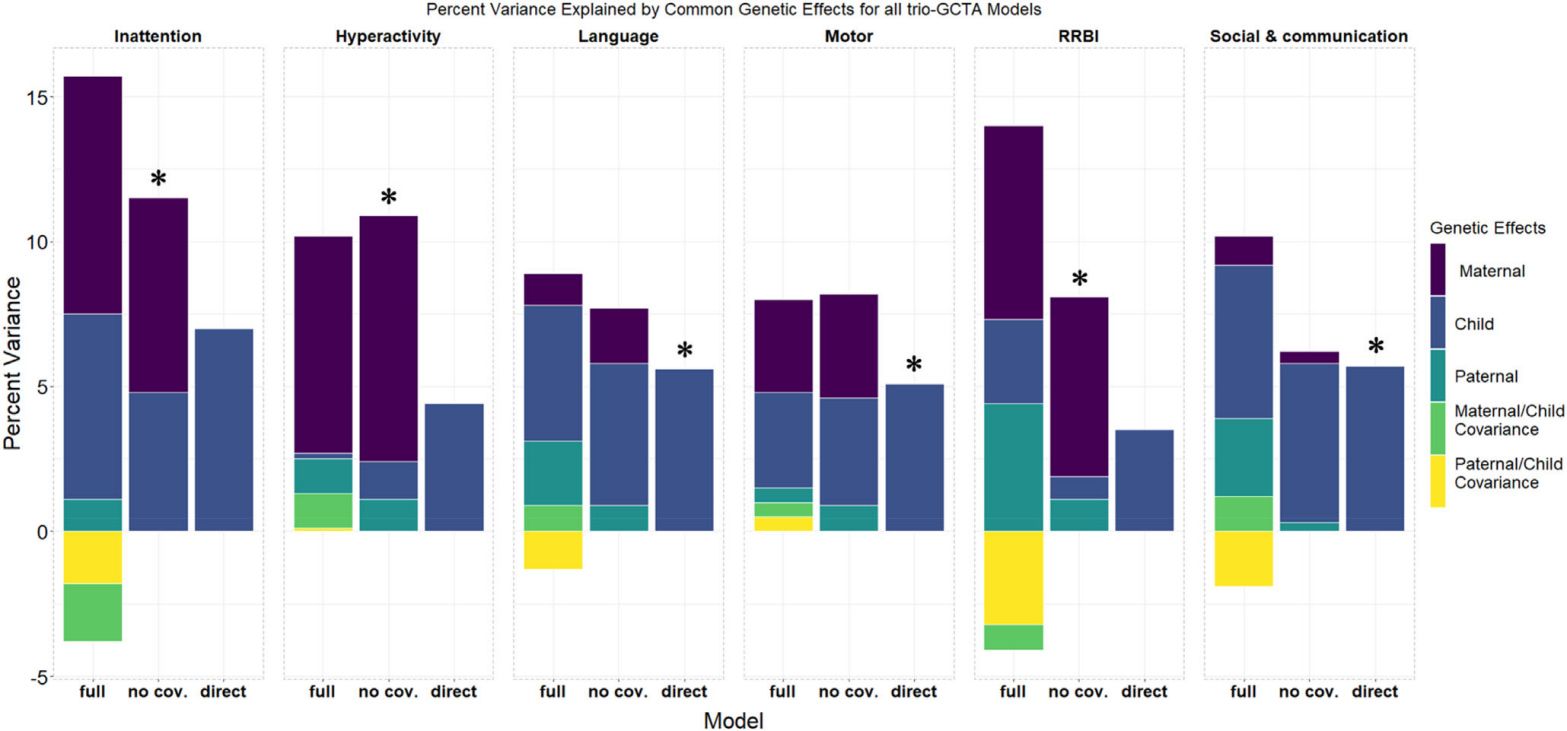
Variance component estimates for the full model estimating all parameters, the model not including covariances parameters (no cov.), and the direct effects only model. The best‐fitting model based on AIC is indicated with a ‘*’. Indirect effects include the variance explained by the genotypes of the mother and father while direct effects are the variance explained by the genotype of the child. In the full models, the covariance between the mother and child's genetic effects and the father and child's genetic effects is estimated (left‐most bar of each panel). This represents the extent to which the same genetic variation contributes to maternal/paternal direct and indirect genetic effects. When this covariance is positive, this will increase the total variability of genetic effects explained in the outcome. However, when the covariance between direct and indirect effects is negative, the true proportion of variance explained in the outcome by direct and indirect genetic effects is reduced as the effects cancel each other out

The estimated variance components for best‐fitting and alternative Trio‐GCTA models including genetic effects are shown in Figure 1. Estimates from the best‐fitting model for each outcome are indicated with an asterisk. For all outcomes, model fit statistics were in favor of simpler models not including covariances between indirect and direct effects.

In the models with the lowest AIC, direct genetic effects accounted for 4.8% [*SE* = 2.4%] of the variance in early inattention, 1.3% [*SE* = 2%] in hyperactivity, 0.8% [*SE* = 2.3%] in RRBI, 5.6% [*SE* = 2%] in language development, 5.1% [*SE* = 2.1%] in motor development, and 5.7% [*SE* = 2%] in social communication traits. Surprisingly, indirect genetic effects, and particularly maternal genetic effects, explained more variance relative to direct effects for hyperactivity (maternal indirect: 8.5% [*SE* = 2.1%], paternal indirect: 1.1% [*SE* = 2.1%]) and RRBI (maternal indirect: 6.2% [*SE* = 2.1%], paternal indirect: 1.1% [*SE* = 2.1%]). For inattention, the magnitude of maternal indirect genetic effects (6.7% [*SE* = 2.7%]) was similar to the estimate for direct genetic effects. Paternal indirect effects did not explain any variability in early inattention. For social communication traits, language, and motor development, models without indirect effects were preferred. A post hoc analysis of an SCQ total score showed direct effect estimates similar to those of social and communication traits and indirect effects similar to RRBI (Tables S7 and S9). Variance component estimates for all models are reported in Table S4.

### Single‐trait trio‐PGS regression models

**Figure 2:**
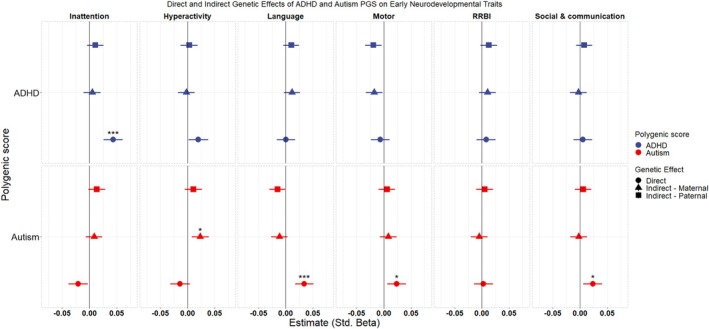
Standardized beta estimates for ADHD and autism PGS on the six measures of early neurodevelopmental traits. And 95% confidence intervals are shown. ‘*’and ‘***’ denote adjusted *p*‐values <.05 and <.001 after multiple testing correction. All results presented are the PGS effect adjusting for the effect of the PGS for the other members of the trio

In the trio‐PGS analyses, we found that direct genetic effects on early neurodevelopmental traits are currently not well captured by PGS of neurodevelopmental conditions. The results for these models for the ADHD and autism PGS are shown in Figure 2. In the main analyses, the only direct genetic effects surviving correction for multiple testing were the ADHD PGS on inattention (*β* = 0.04 [0.03–0.06]) and the autism PGS on language development (*β* = 0.03 [0.02–0.05]), motor development (*β* = 0.02 [0.01–0.04]), and social communication traits (*β* = 0.02 [0.01–0.04]). We did not find any significant effects for the dyslexia PGS (Figure S1). The autism PGS also significantly increased the likelihood of reporting difficulties in both receptive and expressive language development as well as gross (but not fine) motor development (Figure S2).

Indirect effects surviving multiple testing of these PGS were found exclusively in mothers. The autism PGS is associated with reporting more hyperactivity behaviors (*β* = 0.02 [0.01–0.04]). The maternal autism PGS was also associated with a decreased likelihood of reporting receptive language difficulties, but this association was not found for expressive language or the overall language measure.

**Figure 3:**
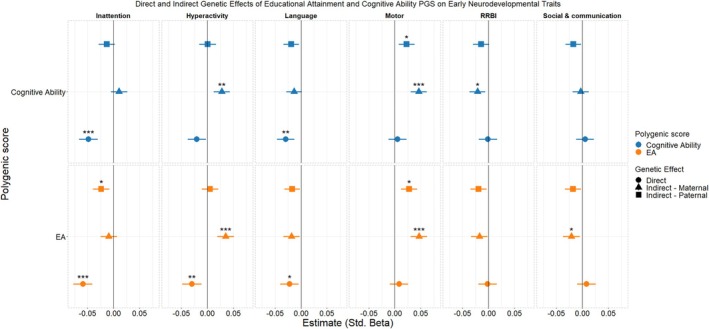
Standardized beta estimates for cognitive ability and educational attainment (EA) PGS on the six measures of early neurodevelopmental traits. 95% confidence intervals are shown. ‘*’, ‘**’, ‘***’ denote adjusted *p*‐values <.05, <.01, and <.001 after multiple testing correction. All results presented are the PGS effect adjusting for the effect of the PGS for the other members of the trio

Figure 3 shows the results for single‐trait trio‐PGS models of early neurodevelopmental traits regressed on cognitive ability and educational attainment PGS. Educational attainment and cognitive ability contributed to both direct and indirect effects on the neurodevelopmental traits. Direct effects surviving multiple testing were observed for both educational attainment (*β* = −0.06 [−0.07 to −0.04]) and cognitive ability (*β* = −0.05 [−0.07 to −0.03]) on inattention and language (EA: *β* = −0.02 [−0.04 to −0.01]; Cog. Ability: *β* = −0.03 [−0.05 to −0.01]), and for educational attainment only on hyperactivity (*β* = −0.03 [−0.05 to −0.01]).

Between them, the educational attainment and cognitive ability PGS indicated indirect effects (either maternal, paternal, or both) on all domains of neurodevelopment except the overall language measure (although indirect effects were found to contribute to reporting increased difficulties in expressive language development) with varying directions of effects. For the motor domain, we found that both maternal and paternal PGS for educational attainment and cognitive ability were significantly positively associated with increased difficulties. When effects were modeled at the level of fine and gross motor subdomains, we observed that the indirect effects on motor development were primarily found for fine motor skills (Figure S2). In addition, a significant positive direct genetic effect of educational attainment on gross motor skill difficulties was present that was not found for the overall motor measure. Model parameters for the single PGS‐trait models are reported in Table S5 (main analyses) and Table S8 (SCQ total score).

### Multi‐trait trio‐PGS regression models

PGS for autism, ADHD, dyslexia, educational attainment, and cognitive ability – included for all members of a family trio – jointly explained very little variance in early neurodevelopmental traits. Together, these scores' direct effects only explain 0.01%–0.27% of the variance, and indirect effects explain 0.06%–0.23% of the variance in the neurodevelopmental traits (Table S6). Sensitivity analyses removing siblings had little effect on the estimates (Table S6). In the multi‐trait models, direct genetic effects explain more variance than indirect genetic effects for inattention and language development while the opposite was true for hyperactivity, motor development, RRBI, and social communication traits, although no formal comparison was made (Table S6).

## Discussion

In this preregistered study, we estimated the relative contributions of direct and indirect genetic effects for a range of maternally reported behavioral traits linked to neurodevelopmental conditions in 3‐year‐old children sampled from the Norwegian population. We did this using two complementary methodological approaches. Using Trio‐GCTA, we found evidence for both direct *and* indirect latent genetic effects on inattention, hyperactivity, and restrictive and repetitive behaviors and interests among 3‐year‐old children, and for direct effects *only* for social communication traits, language, and motor development at the same age. Using a trio‐PGS model, genetic liabilities for ADHD and autism and, inversely, for cognitive ability and educational attainment were specific sources of direct genetic effects, while genetic liabilities for cognitive ability and educational attainment were sources of maternal and/or paternal indirect effects for almost all neurodevelopmental outcomes.

Direct genetic effects, usually implicitly assumed to be the predominant component of heritability estimates, were indeed robustly evident for almost all neurodevelopmental traits in our Trio‐GCTA models. While this was not replicated across all traits in the trio‐PGS analyses, this should do little to undermine our confidence that these effects exist, given the tiny fraction of the SNP heritability (and overall variance) in outcomes that current PGS explain. For example, the source GWAS for the autism PGS is of comparatively smaller sample size than the other traits, and the dyslexia PGS was constructed using a much smaller set of SNPs. Instead, we conclude that the direct effects observed at the genome‐wide level are not well captured by the PGS included in the analyses. Future versions of scores used here based on more progressed GWAS will likely capture a larger proportion of these effects. Additionally, ADHD and dyslexia PGS may have weaker associations than expected (for example, no statistically significant association between ADHD PGS and hyperactivity was found) at this age in comparison to older ages since 3 years is typically prior to the age diagnoses of these conditions are reliably made. Differences between the underlying GWAS traits, populations, and sample sizes mean that the presence and magnitude of effects across different PGS are not easily comparable so little should be read into the comparison of genetic liabilities for neurodevelopmental conditions versus educational attainment and cognitive ability. However, what *is* noteworthy here is that we find both direct and indirect effects on early neurodevelopmental traits are captured by educational attainment and cognition, which is consistent with their substantially attenuated heritabilities in within‐family GWAS (Howe et al., 2022).

We found evidence for indirect genetic effects of surprisingly large relative magnitude for three traits (inattention, hyperactivity, RRBI) in the Trio‐GCTA analyses. These effects either exceeded or equaled direct genetic effects in the same models in all cases. This sits in contrast to results from the same sample, using the same and similar methodologies to investigate 8‐year ADHD traits, where direct effect estimates were invariably at least twice as large as indirect equivalents (Eilertsen et al., 2022; Jami et al., 2023). It should be noted that in both cases, the effects in question are quite imprecisely estimated. Although such a developmental shift would be consistent with a developmental model of decreasing passive gene–environment correlation over time (Scarr & McCartney, 1983), elevated indirect effects – in particular, maternal indirect effects – at early time points are also consistent with a greater influence of maternal (genetically‐influenced) rater bias when children are younger. Rater bias may be a particularly likely mechanism for some counter‐intuitive effects, such as higher maternal (notably, not *paternal*) genetic liability to educational attainment being positively associated with reported hyperactivity traits. However, further work is needed to conclusively determine any of the mechanisms underpinning these results. More generally, we observe relatively low proportions of the variance explained by common genetic effects across all traits in the Trio‐GCTA models.

When comparing the two modeling approaches, we see concordance in the findings for some of the traits. For example, direct effects explain 4.8% of the variance in inattention scores at age 3, and this is partly attributable to genetic liability for ADHD being associated with higher reported inattention, and genetic liabilities for educational attainment and cognitive ability being associated with lower reported inattention. However, we also observe seemingly inconsistent results – for example, no overall indirect genetic effects were detected for motor development in the best‐fitting Trio‐GCTA model, yet maternal and paternal genetic liability for cognitive ability and educational attainment are associated with reporting more difficulties in motor development in the trio‐PGS. Here, a key point should be noted to resolve this apparent inconsistency – and in turn, dampen enthusiasm toward the consistencies outlined above. PGS explain a tiny fraction of variance both collectively and on their own. In partitioning variance into latent genome‐wide genetic effects, the significant effects captured by the PGS are likely too small to influence the ‘overall picture’ assessed by the Trio‐GCTA models. Nonetheless, a PGS‐based approach does offer some increased specificity in terms of identifying particular genetic liabilities with which direct and indirect effects are associated. As such, we propose here that their application in parallel allows for a more complete discussion of direct and indirect effects – at both the macro and micro scale.

Finally, it is important to consider how understanding direct and indirect genetic effects on traits may ultimately be used to benefit individuals with neurodevelopmental conditions and their families. Current genomic methods only explain small proportions of variation, making direct translation – for example, in the form of using polygenic scores to help direct and prioritize care and support in a personalized manner – a very distant prospect. However, as we demonstrate here, even as genomic discovery efforts for neurodevelopmental traits are at an early stage, it is already possible to use them to begin to pick apart direct and indirect mechanisms. For example, our findings suggest that maternal education could be an environmental source of variation influencing reporting of hyperactivity and RRBI unconfounded by the child's own genotype. While it is unclear if this represents a true environmentally mediated genetic effect or captures other confounding effects (i.e., the effect of maternal education genetic liability on rating styles), it does mark the relationship as worthy of further investigation as it could have significant implications for early identification. Given the reliability and relative ease with which genetic variation can be measured, methods such as these are likely to become an increasingly important tool for identifying specific environments with particular relevance to those with neurodevelopmental conditions.

### Limitations

There are some limitations that should be considered in the interpretation of these results. Firstly, we find overall low proportions of direct genetic effects. Both low reliabilities of some of the trait measures and limited endorsement for some traits may have contributed to this pattern. Additionally, to preserve statistical power in the sensitivity analyses using dichotomized outcomes, groups were collapsed into ‘no’ versus ‘any’ difficulties, where a more stringent approach (i.e., ‘yes’ vs. ‘never/rarely’) may have been preferable. Further, while the direct effects we do identify in the trio‐PGS analyses are *more* robust to bias from indirect genetic effects, assortative mating, and population stratification than estimates *not* adjusting for parental scores, they are still observational estimates still best interpreted as associations rather than causal effects per se. This may be particularly relevant where the mechanisms for confounding are dynastic, such as assortative mating in generations prior to the parent generation included here, or structural at the GWAS stage, such as residual population stratification and selection into the population‐based GWAS used to construct the PGS (Veller & Coop, 2024). Additionally, recent work has shown that many of the same biases, such as residual population structure, assortative mating, epigenetic imprinting effects, or indirect effects of siblings, that influence direct effect estimates in population‐based studies will be captured in estimates of indirect effects in both trio‐PGS and trio‐GCTA (Eilertsen et al., 2022; Krätschmer & Robinson, 2024; Nivard et al., 2022; Veller & Coop, 2024). Accordingly, we would caution readers against interpreting indirect effect estimates – including those of relatively large magnitude found in the current study – as solely genetic nurture effects. An important avenue of future work will be decomposing these indirect effect estimates into their constituent parts to reveal the true extent of genetic nurturing.

Also important to the interpretation of these results are potential biases arising from both selection and attrition in MoBa, particularly in relation to factors relevant to the traits in the present study (Biele et al., 2019) – such as the overrepresentation of families with higher educational levels and socioeconomic backgrounds in MoBa. (Vejrup, Magnus, & Magnus, 2022) Additionally, we observed that families of children with neurodevelopmental conditions were lost to follow‐up at higher rates than the rest of the cohort. Finally, these analyses were limited to those of European ancestry, limiting the generalizability of these results.

## Conclusions

Understanding the genetic contributions to early neurodevelopmental traits in a within‐family paradigm can give insights into the pathways of genetic transmission involved in the development of neurodevelopmental conditions. We find direct genetic effects contribute to maternally reported early neurodevelopment traits in 3‐year‐old children, with differing levels of importance across the areas of neurodevelopment. Indirect genetic effects may be particularly relevant to explaining variability in early measures of inattention, hyperactivity, and restricted and repetitive behaviors and interests. Both direct and indirect effects are captured by PGS for neurodevelopmental conditions and related traits, albeit with small effect sizes. Overall, direct and indirect genetic effects influence neurodevelopmental traits at age 3 to varying degrees, highlighting the importance of within‐family approaches for disentangling genetic processes that influence the traits and their relation to neurodevelopmental conditions.

## Ethical considerations

The establishment of MoBa and initial data collection was based on a license from the Norwegian Data Protection Agency and approval from The Regional Committees for Medical and Health Research Ethics. The MoBa cohort is currently regulated by the Norwegian Health Registry Act. The current study was approved by The Regional Committees for Medical and Health Research Ethics (2016/1702).

**Key points**

- Using two complementary methodological approaches, we investigate the relative contributions of direct and indirect genetic effects on mothers' reports about traits linked to neurodevelopmental conditions in 3‐year‐old children sampled from the Norwegian population.
- Direct effects were robustly found for almost all traits. However, indirect effects – where parents' genes are linked to their children's traits independent of the children's own genes – also contributed to explaining variability.
- Polygenic scores for cognitive ability and educational attainment were sources of maternal or paternal indirect effects for most neurodevelopmental traits, while genetic liability for neurodevelopmental conditions conferred indirect genetic effects for only a few outcomes, and only from mothers.

## Supporting information


**Appendix S1.** Supplementary note.
**Appendix S2.** Supplementary methods.
**Figure S1.** Direct and indirect genetic effects of the dyslexia PGS on early neurodevelopmental traits.
**Figure S2.** Direct and indirect genetic effects of all PGS on subdomains of language and motor development.
**Figure S3.** Direct and indirect genetic effects of ADHD and autism PGS on early neurodevelopmental traits using PGS created with PRScise2.
**Figure S4.** Direct and indirect genetic effects of educational attainment and cognitive ability PGS on early neurodevelopmental traits using PGS created with PRScise2.
**Figure S5.** Direct and indirect genetic effects of PGS on subdomains of language and motor development using PGS created with PRScise2.
**Table S1.** Endorsement rates of the subdomains of motor (gross and fine motor) and language (expressive and receptive) development.
**Table S2.** Values on trait measures for individuals with and without genotyping information. Scales presented are standardized to mean of 0 and *SD* of 1.
**Table S3.** PGS values for trio members of children with and without age 3 trait measures.
**Table S4.** Variance component estimates for all Trio‐GCTA models.
**Table S5.** Model parameters for the single PGS‐trait models.
**Table S6.** Variance explained for direct versus indirect effect in multi‐trait trio‐PGS models.
**Table S7.** Trio‐GCTA model fit for a SCQ total score.
**Table S8.** Model parameters for the single PGS‐trait model for a SCQ total score.
**Table S9.** Trio‐GCTA model variance component estimates for a SCQ total score.
**Table S10.** Model parameters for the single PGS‐trait models using PGS created with PRScise2.
**Table S11.** Variance explained for direct versus indirect effect in multi‐trait trio‐PGS models using PGS calculated with PRScise2.

## Data Availability

The consent given by the participants does not allow for storage of data on an individual level in repositories. Researchers can apply for access to data for replication purposes via MoBa, in line with MoBa data access policies. Analytical code for this study can be found at https://github.com/psychgen/within‐fam‐early‐neurodev‐moba. Documentation for MoBa questionnaires can be found at https://www.fhi.no/en/ch/studies/moba/for‐forskere‐artikler/questionnaires‐from‐moba/.
