# Supplementary material for: Direct and indirect genetic effects on early neurodevelopmental traits
Source: J Child Psychol Psychiatry. 2025 Jan 30;66(7):1053–64. doi: 10.1111/jcpp.14122 (PMC12198931; doi:10.1111/jcpp.14122)
Supplement: Supplementary file 1 — Appendix S1. Supplementary note. Appendix S2. Supplementary methods. Figure S1. Direct and indirect genetic effects of the dyslexia PGS on early neurodevelopmental traits. Figure S2. Direct and indirect genetic effects of all PGS on subdomains of language and motor development. Figure S3. Direct and indirect genetic effects of ADHD and autism PGS on early neurodevelopmental traits using PGS created with PRScise2. Figure S4. Direct and indirect genetic effects of educational attainment and cognitive ability PGS on early neurodevelopmental traits using PGS created with PRScise2. Figure S5. Direct and indirect genetic effects of PGS on subdomains of language and motor development using PGS created with PRScise2. Table S1. Endorsement rates of the subdomains of motor (gross and fine motor) and language (expressive and receptive) development. Table S2. Values on trait measures for individuals with and without genotyping information. Scales presented are standardized to mean of 0 and SD of 1. Table S3. PGS values for trio members of children with and without age 3 trait measures. Table S4. Variance component estimates for all Trio‐GCTA models. Table S5. Model parameters for the single PGS‐trait models. Table S6. Variance explained for direct versus indirect effect in multi‐trait trio‐PGS models. Table S7. Trio‐GCTA model fit for a SCQ total score. Table S8. Model parameters for the single PGS‐trait model for a SCQ total score. Table S9. Trio‐GCTA model variance component estimates for a SCQ total score. Table S10. Model parameters for the single PGS‐trait models using PGS created with PRScise2. Table S11. Variance explained for direct versus indirect effect in multi‐trait trio‐PGS models using PGS calculated with PRScise2. [file JCPP-66-1053-s001.docx]

Supplementary material for Direct and Indirect Genetic Effects on Early Neurodevelopmental Traits

Laura Hegemann^1,2,3^, Espen Eilertsen^4^, Johanne Hagen Pettersen^1,3,5^, Elizabeth C. Corfield^2,3^, Rosa Cheesman^4^, Leonard Frach^6^, Ludvig Daae Bjørndal^4^, Helga Ask^3,4^, Beate St Pourcain^7,8,9^, Alexandra Havdahl^2,3,4^_,_ Laurie J. Hannigan^3,2,7^

^1^ Department of Psychology, University of Oslo, Oslo, Norway

^2^ Nic Waals Institute, Lovisenberg Diaconal Hospital, Oslo, Norway

^3^ PsychGen Center for Genetic Epidemiology and Mental Health, Norwegian Institute of Public Health, Oslo, Norway

^4^ PROMENTA Research Center, Department of Psychology, University of Oslo, Oslo,

Norway

^5^Department of Child Health and Development, Norwegian Institute of Public Health, Oslo, Norway.
^6^ Department of Clinical, Educational and Health Psychology, Division of Psychology and Language Sciences, University College London, London, UK.

^7^ MRC Integrative Epidemiology Unit (IEU), University of Bristol, Bristol, United Kingdom

^8^Language and Genetics Department, Max Planck Institute for Psycholinguistics, Nijmegen, the Netherlands

^9^Donders Institute for Brain, Cognition and Behaviour, Radboud University, Nijmegen, the Netherlands

Table of Contents

[Appendix S1: Supplementary Note 3](#_Toc187670789)

[Appendix S2: Supplementary Methods 4](#_Toc187670790)

[Measures 4](#_Toc187670791)

[Polygenic scores 8](#_Toc187670792)

[Supplementary Tables and Figures 10](#_Toc187670793)

[Supplementary Table 1 10](#_Toc187670794)

[Supplementary Table 2 10](#_Toc187670795)

[Supplementary Table 3 11](#_Toc187670796)

[Supplementary Table 4 11](#_Toc187670797)

[Supplementary Figure 1 13](#_Toc187670798)

[Supplementary Figure 2 14](#_Toc187670799)

[Supplementary Table 5 15](#_Toc187670800)

[Supplementary Table 6 22](#_Toc187670801)

[Results for post-hoc analyses using SCQ total score: 23](#_Toc187670802)

[Supplementary table 7: trio-GCTA model fit 23](#_Toc187670803)

[Supplementary table 8: Model parameters for the single PGS-trait model 23](#_Toc187670804)

[Supplementary table 9: trio-GCTA model variance component estimates 24](#_Toc187670805)

[Original Results using PGS created in PRSice2 using the pre-registered method 25](#_Toc187670806)

[Supplementary Figure 3: Direct and Indirect Genetic Effects of ADHD and autism PGS on Early Neurodevelopmental Traits 26](#_Toc187670807)

[Supplementary Figure 4: Direct and Indirect Genetic Effects of Educational Attainment and Cognitive Ability PGS on Early Neurodevelopmental Traits 27](#_Toc187670808)

[Supplementary Figure 5: Direct and Indirect Genetic Effects of PGS on subdomains of Language and Motor Development 28](#_Toc187670809)

[Supplementary Table 10: Model parameters for the single PGS-trait models 29](#_Toc187670810)

[Supplementary Table 11: Variance explained for direct vs. indirect effect in multi-trait trio-PGS models 37](#_Toc187670811)

[Supplementary References 38](#_Toc187670812)

# **Appendix S1: Supplementary Note**

*Note on deviations from the pre-registration*

Due to constraints on analytic possibilities resulting from the observed distributions of the data, we were forced to deviate from the preregistered analysis plan. The original pre-registration can be found on the OSF repository here: <https://osf.io/zwk2a> and the updated document here: <https://osf.io/xjfw9>

An overview of the reasoning behind the deviations:

**Removal of bifactor model:**  The original version of the preregistration for this study included modeling general and domain-specific factors underlying early neurodevelopmental traits in an SEM framework, using the items from the scales as indicators of these factors. However, the initial investigation revealed that both the planned broad version (four domain-specific factors) and narrow version (eight domain-specific factors) of these bifactor models provided a poor fit to the data with substantial evidence for model misspecification. Exploratory factor analysis that included an expanded set of items from those included in this analysis found that a correlated factor model was a substantially better way of explaining covariance between early neurodevelopmental items, indicating a large amount of heterogeneity underlying these traits in the general population and little evidence for a shared general factor (Hegemann et al., 2024). Therefore, instead of proceeding with a misspecified model we chose to remove the bifactor model from the analysis. Because of the indication of many factors underlying these early traits in general populations we chose to move forward breaking the scales into subscales in accordance with our original “narrow” version of the specific factors. Allowing us to look at estimates of genetic effects with more specificity than using the “broad” version. Because of the distributions of the motor and language subscales, we further deviated from this. We used the “broad” version of the motor and language subscales in order to have a continuous measure of these domains for the trio-GCTA analyses and, for consistency, used these in addition to the “narrow” versions in the trio-PGS analyses.

**Use of observed scores over factor scores:** Because of the removal of the bifactor model there was no longer the need for estimating general and specific components underlying variance in early neurodevelopmental traits from the items so observed scores could be used instead.

**Removal of the general and specific measurement model analyses:** Due to the poor fit and evidence of misspecification of the bifactor model we concluded that it was not feasible to move forward with further models based on it.

**Changes to source GWAS PGS:** The ADHD and dyslexia PGS were created with the more recent GWAS for both traits that had become available after the initial pre-registration. A PGS created from the UK biobank handgrip measure was dropped from the updated pre-registration, prior to running to the trio-PGS models. Originally the score was included under the logic that it was the best proxy available for motor development. However, under further consideration we concluded that the PGS analyses should be limited to PGS for neurodevelopmental conditions and traits that have substantial literature backing there associations to these conditions.

**Change to method to calculate PGS:** As per recommendation of a reviewer we changed the method to calculate PGS to a more updated method than the method we had specified in the preregistration. We switched to using the LDpred2 software which incorporates SNP-based heritability and polygenicity into a Bayesian model to estimate scores (Privé et al., 2021). This was as opposed to our original method were scores were calucated for a range of p-value thresholds and the first principal component from a principal component analysis of the resultant set of scores was used. The orginal results using these scores can be found here.

# **Appendix S2: Supplementary Methods**

## Measures

The MoBa questionnaire included two items from both the fine and gross motor subscales, which were combined to form an overall measure of motor development. Six items covering receptive and expressive language development (three items each) from the ASQ communication subscale were combined as a measure of language development.

In sensitivity analyses, the fine motor and expressive language development subscales were treated as ordinal while receptive language and gross motor development were dichotomized so that all values greater than 0 were coded as 1 and interpreted as no versus any reported difficulties in these areas.

For remaining individuals, the sum score was divided by the number of items answered and multiplied by the total number of items in the total score. This value rounded to the nearest whole number was presented as the total/subdomain scores.

Full instrument documentation on the MoBa questionnaires can be found elsewhere: <https://www.fhi.no/en/ch/studies/moba/for-forskere-artikler/questionnaires-from-moba/#36-months-after-birth>

The following instruments where used for the measures of early neurodevelopmental traits:

| Outcome | Instrument | Items |
| --- | --- | --- |
| Social & communication | SCQ | Does your child ever talk with you just to be friendly (rather than to get something)?  Do you have a to and fro "conversation" with her/him that involves taking turns or building on what you have said?  Does your child ever use socially inappropriate questions or statements?  Does your child’s facial expression usually seem appropriate to the particular situation, as far as you can tell?  Does your child ever use your hand like a tool or as if it were part of his/her own body?  Does your child have any particular friends or a best friend?  Does your child ever spontaneously copy you (or other people) or what you are doing?  Does your child ever spontaneously point at things around him/her just to show you things (not because he/she wants them)?  Does your child ever use gestures, other than pointing or pulling your hand, to let you know what he/she wants?  Does your child nod his/her head to indicate yes?  Does your child shake his/her head to indicate no?  Does your child usually look at you directly in the face when doing things with you or talking with you?  Does your child smile back if someone smiles at him/her?  Does your child ever show you things that interest him/her to engage your attention?  Does your child ever offer to share things other than food with you?  Does your child ever seem to want you to join in his/her enjoyment of something?  Does your child ever try to comfort you when you are sad or hurt?  If your child wants something or wants help, does he/she look at you and use gestures with sounds or words to get your attention?  Does your child show a normal range of facial expressions?  Does your child ever spontaneously join in and try to copy the actions in social games?  Does your child seem interested in other children of approximately the same age whom he/she does not know?  Does your child respond positively when another child approaches him/her?  If you come into a room and start talking to your child without calling his/her name, does he/she usually look up and pay attention to you?  Does your child ever play imaginative games with another child in such a way that you can tell that each child understands what the other is pretending?  Does your child play cooperatively in games that need some form of joining in with a group of other children? |
| RRBI | SCQ | Does she/he ever use odd phrases or say the same thing over and over in almost exactly the same way?  Does your child ever get his/her pronouns mixed up?  Does your child ever use words that he/she seems to have invented or made up her/himself; put things in odd, indirect ways; or use metaphorical ways of saying things (e.g., saying hot rain for steam)?  Does your child ever say the same thing over and over in exactly the same way or insist that you say the same thing over and over again?  Does your child ever have any interests that preoccupy him/her and might seem odd to other people?  Does your child ever seem to be more interested in parts of a toy or an object (e.g., spinning the wheels of a car), rather than in using the object as it was intended?  Does your child ever have any special interests that are unusual in their intensity, but otherwise appropriate for his/her age and peer group (e.g., trains or dinosaurs)?  Does your child ever seem to be unusually interested in the sight, feel, sound, taste, or smell of things or people?  Does your child ever have any mannerisms or odd ways of moving his/her hands or fingers, such as flapping or moving his/her fingers in front of his/her eyes?  Does your child ever have any complicated movements of his/her whole body, such as spinning or repeatedly bouncing up and down? |
| inattention | CBCL | Can’t concentrate, can’t pay attention for long  Can’t sit still, restless or overactive  Quickly shifts from one activity to another |
| Hyperactivity | CBCL | Can’t stand waiting, wants everything now  Demands must be met immediately  Gets into everything |
| Motor | ASQ – Motor | Without holding onto anything for support, does your child kick a ball by swinging his/her leg forward?  Can your child catch a large ball with both hands?  When drawing, does your child hold a pencil, crayon, or pen between his/her fingers and thumb like an adult does?  Can your child undo one or more buttons? |
| Language | ASQ – Communication | Without showing him/her first, does your child point to the correct picture when you say, “Where is the cat” or “Where is the dog”?  When you ask your child to point to his/her eyes, nose, hair, feet, ears, and so forth, does he/she correctly point to at least seven body parts?  Does your child make sentences that are three or four words long?  Without giving him/her help by pointing or using gestures, ask your child to “Put the shoe on the table” and “Put the book under the chair”. Does your child carry out both of these directions correctly?  When looking at a picture book, does your child tell you what is happening or what action is taking place in the picture?  Can your child tell you at least two things about an object he/she is familiar with? |
| Fine Motor | ASQ – Motor | When drawing, does your child hold a pencil, crayon, or pen between his/her fingers and thumb like an adult does?  Can your child undo one or more buttons? |
| Gross Motor | ASQ – Motor | Without holding onto anything for support, does your child kick a ball by swinging his/her leg forward?  Can your child catch a large ball with both hands? |
| Expressive Language | ASQ – Communication | Does your child make sentences that are three or four words long?  When looking at a picture book, does your child tell you what is happening or what action is taking place in the picture?  Can your child tell you at least two things about an object he/she is familiar with? |
| Receptive Language | ASQ – Communication | Without showing him/her first, does your child point to the correct picture when you say, “Where is the cat” or “Where is the dog”?  When you ask your child to point to his/her eyes, nose, hair, feet, ears, and so forth, does he/she correctly point to at least seven body parts?  Without giving him/her help by pointing or using gestures, ask your child to “Put the shoe on the table” and “Put the book under the chair”. Does your child carry out both of these directions correctly? |

## Polygenic scores

Polygenic scores (PGS) used in the main text were calculated using the software LDpred2 (Privé et al., 2021). We followed an existing workflow that can be found here: <https://github.com/AndreAllegrini/LDpred2?tab=readme-ov-file>. This was based on the recommend QC outline here: <https://privefl.github.io/bigsnpr/articles/LDpred2.html> and in Privé et al. (2022). Precomputed LD matrices from UK Biobank were used as the reference LD panel found here: <https://figshare.com/articles/dataset/LD_reference_for_HapMap3_/21305061> from Privé et al. (2023)

The pre-registration for this paper outlined the following for creation of the polygenic scores:

(PGS) were estimated with the software PRSice2 (Choi & O’Reilly, 2019). PGS for each of the five traits were derived based on creating scores at 11 different p-value thresholds between 5 × 10^-8^ and 1, then extracting the first principal component of these 11 scores for use as the PGS for that trait in the subsequent analyses. This approach controls type one error rate while still maintaining prediction performance (Coombes et al., 2020). PGS were regressed on the first 10 genomic principal components (PCs) and genotype batch.

Results of the analyses using these PGS are presented on page 27

# **Supplementary Tables and Figures**

Supplementary Table 1: Endorsement rates of the subdomains of motor (gross and fine motor) and language (expressive and receptive) development

| Measure | N | N - No Difficulties | N – Any Difficulties | N – score 0 | N – score 1 | N – score 2 | N – score  3 | N – score 4 | N – score 5 | N – score 6 | Median | Regression Type |
| --- | --- | --- | --- | --- | --- | --- | --- | --- | --- | --- | --- | --- |
| Gross motor | 24586 | 19337 | 5249 | 19337 | 4450 | 715 | 51 | 33 |  |  | 0 | Logistic |
| Receptive Language | 24601 | 23090 | 1511 | 23090 | 1245 | 178 | 34 | 21 | 14 | 19 | 0 | Logistic |
| Fine Motor | 24442 | N/A | N/A | 11895 | 5921 | 4015 | 1730 | 881 |  |  | 1 | Ordinal |
| Expressive  Language | 24578 | N/A | N/A | 15417 | 6455 | 1887 | 483 | 150 | 79 | 107 | 0 | Ordinal |

N no difficulties / N any difficulties – number of individuals for each value of the outcome if the measure was dichotomized for logistic regression. N score – number of individuals with that value of the measure.

Supplementary Table 2**:** Values on trait measures for individuals with and without genotyping information. Scales presented are standardized to mean of 0 and SD of 1

| **Scale** | **Values on trait measures for genotyped children** | **Values on trait measures for children not genotyped** | **T test**  **padj** |
| --- | --- | --- | --- |
| Inattention | -0.015 | 0.04 | 0 |
| Hyperactivity | -0.006 | 0.016 | 0.023 |
| Motor | -0.006 | 0.015 | 0.116 |
| Language | -0.02 | 0.056 | 0 |
| RRBI | -0.024 | 0.066 | 0 |
| Social Communication | -0.014 | 0.04 | 0 |

T test Padj – adjusted p value correction (FDR) from a two-sided t-test.

Supplementary Table 3**:** PGS values for trio members of children with and without age 3 trait measures.

| **PGS** | **trio member** | **genotyped w/ age 3** | **genotyped w/o age 3** | **T test padj** |
| --- | --- | --- | --- | --- |
| ADHD | Child | 1.106 | 1.157 | 0 |
| Autism | Child | 1.223 | 1.224 | 0.749 |
| Cog. ability | Child | -0.211 | -0.242 | 0 |
| EA | Child | -0.472 | -0.507 | 0 |
| Dyslexia | Child | -0.424 | -0.417 | 0 |
| ADHD | Mother | 1.099 | 1.152 | 0 |
| Autism | Mother | 1.231 | 1.227 | 0.115 |
| Cog. ability | Mother | -0.202 | -0.237 | 0 |
| EA | Mother | -0.465 | -0.501 | 0 |
| Dyslexia | Mother | -0.426 | -0.42 | 0 |
| ADHD | Father | 1.106 | 1.133 | 0 |
| Autism | Father | 1.216 | 1.219 | 0.452 |
| Cog. ability | Father | -0.214 | -0.23 | 0 |
| EA | Father | -0.472 | -0.495 | 0 |
| Dyslexia | Father | -0.422 | -0.42 | 0.509 |

T test Padj – adjusted p value correction (FDR) from a two-sided t-test.

Supplementary Table 4: Variance component estimates for all Trio-GCTA models

| model | outcome | o | m | p | om | op | mp | e | Lowest AIC |
| --- | --- | --- | --- | --- | --- | --- | --- | --- | --- |
| full | Inattention | 0.064 | 0.082 | 0.011 | -0.02 | -0.018 | 0.028 | 0.88 | no |
| nocov | Inattention | 0.048 | 0.067 | 0 | 0 | 0 | 0 | 0.885 | yes |
| direct | Inattention | 0.07 | 0 | 0 | 0 | 0 | 0 | 0.93 | no |
| null | Inattention | 0 | 0 | 0 | 0 | 0 | 0 | 1 | no |
| full | Hyperactivity | 0.002 | 0.075 | 0.012 | 0.012 | 0.001 | 0.01 | 0.898 | no |
| nocov | Hyperactivity | 0.013 | 0.085 | 0.011 | 0 | 0 | 0 | 0.891 | yes |
| direct | Hyperactivity | 0.044 | 0 | 0 | 0 | 0 | 0 | 0.956 | no |
| null | Hyperactivity | 0 | 0 | 0 | 0 | 0 | 0 | 1 | no |
| full | RRBI | 0.029 | 0.067 | 0.044 | -0.009 | -0.032 | 0.034 | 0.902 | no |
| nocov | RRBI | 0.008 | 0.062 | 0.011 | 0 | 0 | 0 | 0.918 | yes |
| direct | RRBI | 0.035 | 0 | 0 | 0 | 0 | 0 | 0.965 | no |
| null | RRBI | 0 | 0 | 0 | 0 | 0 | 0 | 1 | no |
| full | Social & communication | 0.053 | 0.01 | 0.027 | 0.012 | -0.019 | 0.008 | 0.917 | no |
| nocov | Social & communication | 0.055 | 0.004 | 0.003 | 0 | 0 | 0 | 0.938 | no |
| direct | Social & communication | 0.057 | 0 | 0 | 0 | 0 | 0 | 0.943 | yes |
| null | Social & communication | 0 | 0 | 0 | 0 | 0 | 0 | 1 | no |
| full | Language | 0.047 | 0.011 | 0.022 | 0.009 | -0.013 | 0.011 | 0.924 | no |
| nocov | Language | 0.049 | 0.019 | 0.009 | 0 | 0 | 0 | 0.923 | no |
| direct | Language | 0.056 | 0 | 0 | 0 | 0 | 0 | 0.944 | yes |
| null | Language | 0 | 0 | 0 | 0 | 0 | 0 | 1 | no |
| full | Motor | 0.033 | 0.032 | 0.005 | 0.005 | 0.005 | -0.008 | 0.919 | no |
| nocov | Motor | 0.037 | 0.036 | 0.009 | 0 | 0 | 0 | 0.918 | no |
| direct | Motor | 0.051 | 0 | 0 | 0 | 0 | 0 | 0.949 | yes |
| Null | Motor | 0 | 0 | 0 | 0 | 0 | 0 | 1 | no |

o – variance offspring. m -variance mother. p – variance father. om -covariance between child and mother . op – covariance between child and father. mp – covariance between mother and father. e – residual variance

**
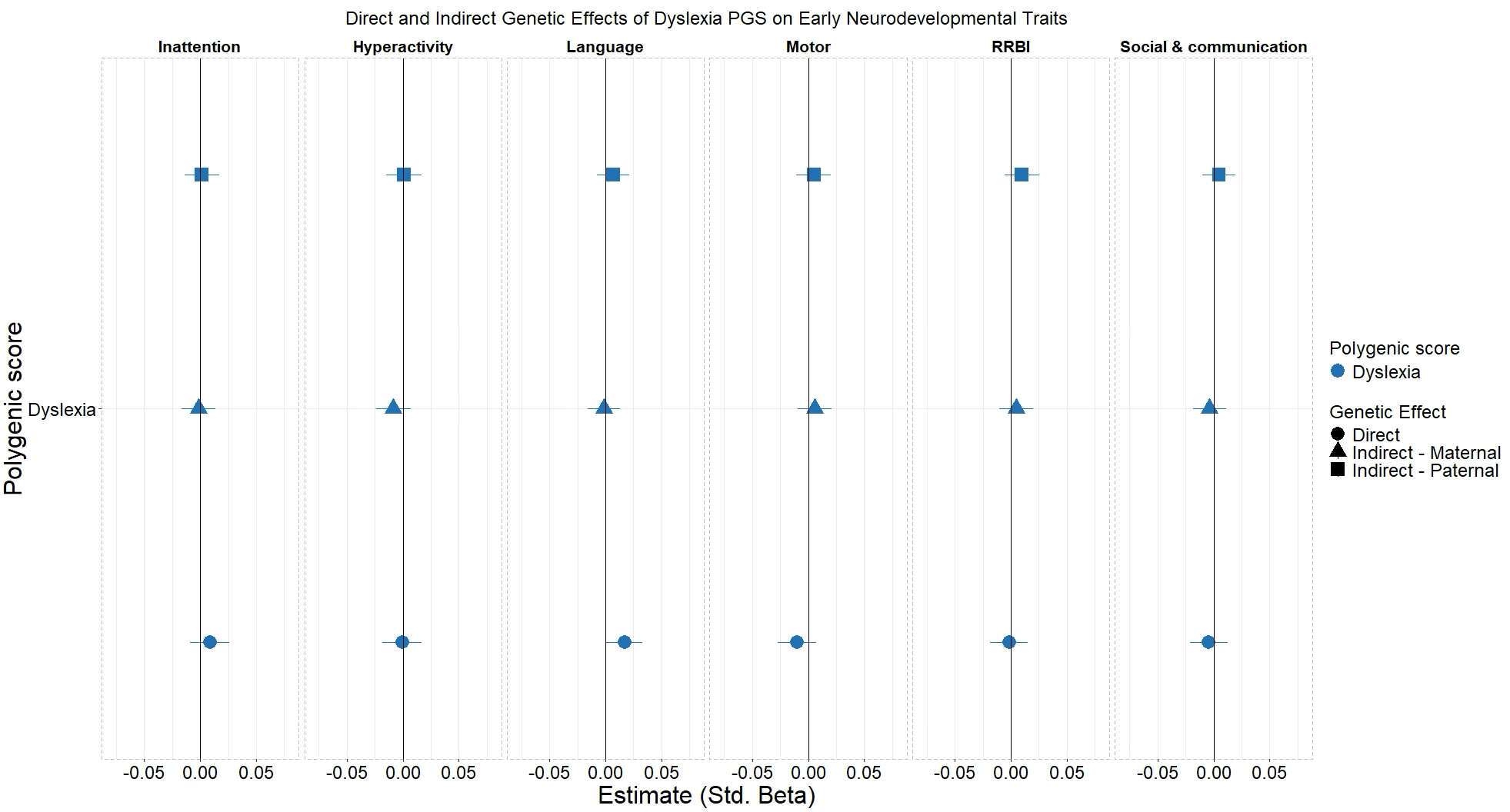
**Supplementary Figure 1: Direct and Indirect Genetic Effects of the Dyslexia PGS on Early Neurodevelopmental Traits

Figure S1: Standardized beta estimates for dyslexia PGS on the six measures of early neurodevelopmental traits. 95% confidence intervals are shown. “*”, “**”, “***” denote adjusted p-values <0.05, <0.01, and <0.001 after multiple testing correction. All results presented are the PGS effect adjusting for the effect of the PGS for the other members of the trio.

Supplementary Figure 2: Direct and Indirect Genetic Effects of all PGS on subdomains of Language and Motor Development

**
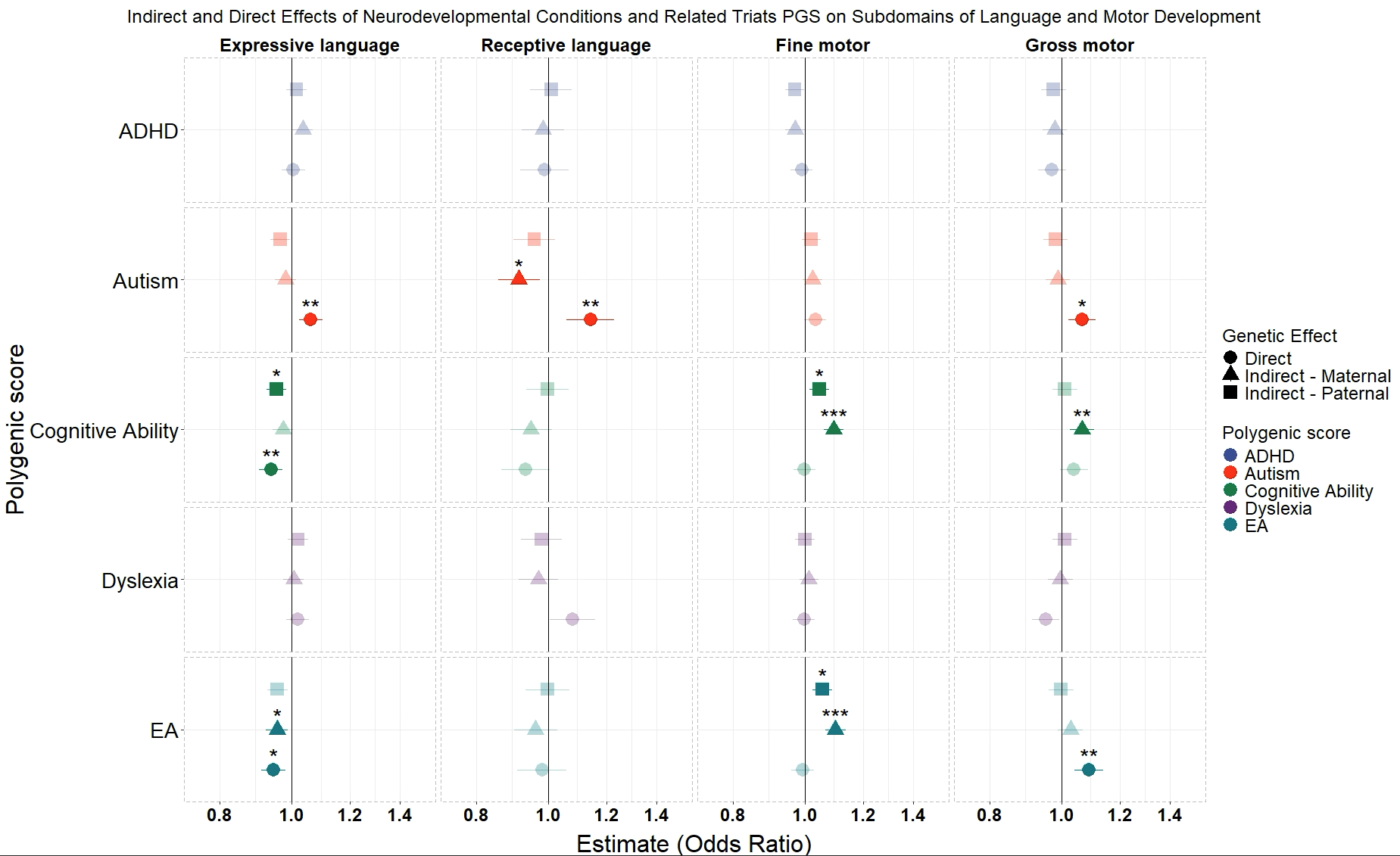
**

Figure S2: estimated log odds ratio for all PGS on likelihood of reporting difficulties in the subdomains of language and motor difficulties. 95% confidence intervals are shown. “*”, “**”, “***” denote adjusted p-values <0.05, <0.01, and <0.001 after multiple testing correction. All results presented are the PGS effect adjusting for the effect of the PGS for the other members of the trio.

Supplementary Table 5: Model parameters for the single PGS-trait models

| **outcome** | **predictor** | **effect** | **Estimate** | **higher_ci** | **lower_ci** | **std.error** | **z or t value** | **p.value.adj** | **sig after MT** |
| --- | --- | --- | --- | --- | --- | --- | --- | --- | --- |
| Inattention | Autism | Direct | -0.02096 | -0.0031779 | -0.0387323 | 0.00906999 | -2.310377 | 0.065236 | FALSE |
| Inattention | Autism | Indirect - Paternal | 0.013341 | 0.0287915 | -0.0021095 | 0.0078829 | 1.692396 | 0.249729 | FALSE |
| Inattention | Autism | Indirect - Maternal | 0.008469 | 0.0237897 | -0.006852 | 0.00781676 | 1.083424 | 0.448018 | FALSE |
| Inattention | ADHD | Direct | 0.04375 | 0.0614964 | 0.026003 | 0.00905444 | 4.831852 | 2.27E-05 | TRUE |
| Inattention | ADHD | Indirect - Paternal | 0.01085 | 0.0261458 | -0.004446 | 0.00780405 | 1.390291 | 0.370832 | FALSE |
| Inattention | ADHD | Indirect - Maternal | 0.005049 | 0.0205891 | -0.0104908 | 0.00792855 | 0.636827 | 0.706383 | FALSE |
| Inattention | EA | Direct | -0.05909 | -0.0407977 | -0.077379 | 0.00933196 | -6.33183 | 1.23E-08 | TRUE |
| Inattention | EA | Indirect - Paternal | -0.02392 | -0.0082533 | -0.0395951 | 0.00799537 | -2.992256 | 0.033525 | TRUE |
| Inattention | EA | Indirect - Maternal | -0.00923 | 0.0066824 | -0.0251392 | 0.00811774 | -1.136821 | 0.448018 | FALSE |
| Inattention | Cognitive Ability | Direct | -0.04814 | -0.0302156 | -0.0660644 | 0.0091451 | -5.264023 | 3.55E-06 | TRUE |
| Inattention | Cognitive Ability | Indirect - Paternal | -0.01319 | 0.002483 | -0.0288575 | 0.00799503 | -1.649432 | 0.249729 | FALSE |
| Inattention | Cognitive Ability | Indirect - Maternal | 0.010638 | 0.0264724 | -0.0051959 | 0.00807864 | 1.316834 | 0.361358 | FALSE |
| Inattention | Dyslexia | Direct | 0.008586 | 0.025908 | -0.0087354 | 0.00883761 | 0.971566 | 0.571166 | FALSE |
| Inattention | Dyslexia | Indirect - Maternal | -0.00155 | 0.0136297 | -0.0167345 | 0.00774597 | -0.200416 | 0.873382 | FALSE |
| Inattention | Dyslexia | Indirect - Paternal | 0.001376 | 0.0167947 | -0.0140431 | 0.00786679 | 0.174881 | 0.977487 | FALSE |
| Hyperactivity | Autism | Direct | -0.01381 | 0.0040666 | -0.0316855 | 0.00912043 | -1.514125 | 0.260014 | FALSE |
| Hyperactivity | Autism | Indirect - Paternal | 0.010809 | 0.0265142 | -0.004897 | 0.00801306 | 1.34887 | 0.370832 | FALSE |
| Hyperactivity | Autism | Indirect - Maternal | 0.023306 | 0.0390128 | 0.00759891 | 0.00801374 | 2.908235 | 0.022738 | TRUE |
| Hyperactivity | ADHD | Direct | 0.01991 | 0.0377814 | 0.00203918 | 0.00911792 | 2.183645 | 0.07631 | FALSE |
| Hyperactivity | ADHD | Indirect - Paternal | 0.00307 | 0.0186738 | -0.0125345 | 0.00796131 | 0.385569 | 0.833118 | FALSE |
| Hyperactivity | ADHD | Indirect - Maternal | -0.00211 | 0.0134078 | -0.0176233 | 0.0079161 | -0.266264 | 0.840466 | FALSE |
| Hyperactivity | EA | Direct | -0.02953 | -0.0110702 | -0.0479823 | 0.00941634 | -3.13564 | 0.008584 | TRUE |
| Hyperactivity | EA | Indirect - Paternal | 0.005321 | 0.0212183 | -0.0105772 | 0.0081111 | 0.655957 | 0.679086 | FALSE |
| Hyperactivity | EA | Indirect - Maternal | 0.035298 | 0.0513205 | 0.01927586 | 0.00817466 | 4.318003 | 0.000158 | TRUE |
| Hyperactivity | Cognitive Ability | Direct | -0.02017 | -0.0023442 | -0.0379923 | 0.00909391 | -2.217778 | 0.073831 | FALSE |
| Hyperactivity | Cognitive Ability | Indirect - Paternal | 0.000656 | 0.0164649 | -0.0151519 | 0.0080655 | 0.081393 | 0.977487 | FALSE |
| Hyperactivity | Cognitive Ability | Indirect - Maternal | 0.027815 | 0.0434483 | 0.01218113 | 0.00797633 | 3.487159 | 0.004075 | TRUE |
| Hyperactivity | Dyslexia | Direct | -0.00072 | 0.016734 | -0.0181681 | 0.00890361 | -0.080536 | 0.957208 | FALSE |
| Hyperactivity | Dyslexia | Indirect - Maternal | -0.0084 | 0.0071134 | -0.0239158 | 0.00791562 | -1.061347 | 0.448018 | FALSE |
| Hyperactivity | Dyslexia | Indirect - Paternal | 0.000855 | 0.0164228 | -0.0147127 | 0.00794274 | 0.107655 | 0.977487 | FALSE |
| Motor | Autism | Direct | 0.023791 | 0.0409793 | 0.00660208 | 0.00876971 | 2.712827 | 0.025676 | TRUE |
| Motor | Autism | Indirect - Paternal | 0.005877 | 0.0209943 | -0.0092407 | 0.00771302 | 0.76193 | 0.675923 | FALSE |
| Motor | Autism | Indirect - Maternal | 0.008245 | 0.0236532 | -0.0071633 | 0.00786134 | 1.048799 | 0.448018 | FALSE |
| Motor | ADHD | Direct | -0.00634 | 0.0108271 | -0.0235009 | 0.00875714 | -0.723626 | 0.711064 | FALSE |
| Motor | ADHD | Indirect - Paternal | -0.019 | -0.003991 | -0.034005 | 0.00765662 | -2.481252 | 0.072771 | FALSE |
| Motor | ADHD | Indirect - Maternal | -0.01767 | -0.0022997 | -0.0330319 | 0.00783984 | -2.253339 | 0.086595 | FALSE |
| Motor | EA | Direct | 0.008272 | 0.0260464 | -0.0095018 | 0.0090684 | 0.912211 | 0.583838 | FALSE |
| Motor | EA | Indirect - Paternal | 0.027449 | 0.0428417 | 0.01205625 | 0.00785342 | 3.495159 | 0.014417 | TRUE |
| Motor | EA | Indirect - Maternal | 0.046552 | 0.0623624 | 0.03074212 | 0.00806639 | 5.771136 | 1.33E-07 | TRUE |
| Motor | Cognitive Ability | Direct | 0.005196 | 0.0224122 | -0.01202 | 0.00878373 | 0.591559 | 0.769655 | FALSE |
| Motor | Cognitive Ability | Indirect - Paternal | 0.022796 | 0.0380053 | 0.00758709 | 0.00775974 | 2.93775 | 0.033525 | TRUE |
| Motor | Cognitive Ability | Indirect - Maternal | 0.046043 | 0.061402 | 0.03068458 | 0.00783609 | 5.875804 | 1.07E-07 | TRUE |
| Motor | Dyslexia | Direct | -0.01013 | 0.0068969 | -0.0271486 | 0.00868507 | -1.165889 | 0.451242 | FALSE |
| Motor | Dyslexia | Indirect - Maternal | 0.006048 | 0.0212262 | -0.0091305 | 0.00774406 | 0.780973 | 0.603925 | FALSE |
| Motor | Dyslexia | Indirect - Paternal | 0.004939 | 0.0202841 | -0.0104069 | 0.00782934 | 0.630784 | 0.679086 | FALSE |
| Fine motor | Autism | Direct | 0.032888 | 0.0670252 | -0.0012485 | 0.01741675 | 1.888317 | 0.13408 | FALSE |
| Fine motor | Autism | Indirect - Paternal | 0.020389 | 0.0504037 | -0.0096258 | 0.01531367 | 1.331422 | 0.370832 | FALSE |
| Fine motor | Autism | Indirect - Maternal | 0.025018 | 0.0555098 | -0.0054738 | 0.01555704 | 1.608144 | 0.265283 | FALSE |
| Fine motor | ADHD | Direct | -0.00912 | 0.0250315 | -0.0432747 | 0.01742505 | -0.523478 | 0.803978 | FALSE |
| Fine motor | ADHD | Indirect - Paternal | -0.03127 | -0.0013781 | -0.0611648 | 0.0152517 | -2.050358 | 0.128864 | FALSE |
| Fine motor | ADHD | Indirect - Maternal | -0.0309 | -0.0005465 | -0.0612489 | 0.01548531 | -1.995293 | 0.135359 | FALSE |
| Fine motor | EA | Direct | -0.00646 | 0.0291635 | -0.0420822 | 0.01817493 | -0.355398 | 0.889133 | FALSE |
| Fine motor | EA | Indirect - Paternal | 0.054003 | 0.0847475 | 0.02325912 | 0.0156858 | 3.442813 | 0.014417 | TRUE |
| Fine motor | EA | Indirect - Maternal | 0.095717 | 0.1270998 | 0.06433422 | 0.01601162 | 5.977972 | 1.07E-07 | TRUE |
| Fine motor | Cognitive Ability | Direct | -0.00062 | 0.0339441 | -0.0351761 | 0.01763271 | -0.034935 | 0.972132 | FALSE |
| Fine motor | Cognitive Ability | Indirect - Paternal | 0.044659 | 0.0749341 | 0.01438471 | 0.01544628 | 2.891274 | 0.033525 | TRUE |
| Fine motor | Cognitive Ability | Indirect - Maternal | 0.089252 | 0.1198304 | 0.05867401 | 0.01560112 | 5.720884 | 1.34E-07 | TRUE |
| Fine motor | Dyslexia | Direct | -0.00234 | 0.0315356 | -0.0362059 | 0.01728098 | -0.135128 | 0.956288 | FALSE |
| Fine motor | Dyslexia | Indirect - Maternal | 0.013207 | 0.0433027 | -0.0168889 | 0.015355 | 0.860102 | 0.556773 | FALSE |
| Fine motor | Dyslexia | Indirect - Paternal | -2.1E-05 | 0.0304364 | -0.0304789 | 0.01553963 | -0.001367 | 0.998909 | FALSE |
| Gross motor | Autism | Direct | 0.064404 | 0.1073583 | 0.02144903 | 0.02191564 | 2.938709 | 0.014981 | TRUE |
| Gross motor | Autism | Indirect - Paternal | -0.01804 | 0.02004 | -0.0561278 | 0.01943055 | -0.928636 | 0.56948 | FALSE |
| Gross motor | Autism | Indirect - Maternal | -0.01033 | 0.027721 | -0.0483795 | 0.0194134 | -0.53207 | 0.743347 | FALSE |
| Gross motor | ADHD | Direct | -0.0292 | 0.0141957 | -0.0725909 | 0.02213943 | -1.318805 | 0.360066 | FALSE |
| Gross motor | ADHD | Indirect - Paternal | -0.02437 | 0.0136769 | -0.06242 | 0.01941249 | -1.255456 | 0.384522 | FALSE |
| Gross motor | ADHD | Indirect - Maternal | -0.02053 | 0.0179464 | -0.058999 | 0.01962894 | -1.045716 | 0.448018 | FALSE |
| Gross motor | EA | Direct | 0.084873 | 0.1301245 | 0.0396211 | 0.0230876 | 3.67612 | 0.002368 | TRUE |
| Gross motor | EA | Indirect - Paternal | -0.00109 | 0.0381984 | -0.0403744 | 0.02004408 | -0.054279 | 0.977487 | FALSE |
| Gross motor | EA | Indirect - Maternal | 0.02778 | 0.0675774 | -0.0120167 | 0.02030462 | 1.368179 | 0.356783 | FALSE |
| Gross motor | Cognitive Ability | Direct | 0.038876 | 0.0827277 | -0.0049749 | 0.02237313 | 1.737638 | 0.171405 | FALSE |
| Gross motor | Cognitive Ability | Indirect - Paternal | 0.010983 | 0.0495017 | -0.0275347 | 0.01965214 | 0.558895 | 0.702723 | FALSE |
| Gross motor | Cognitive Ability | Indirect - Maternal | 0.064099 | 0.1027189 | 0.02547869 | 0.01970412 | 3.253064 | 0.008155 | TRUE |
| Gross motor | Dyslexia | Direct | -0.0493 | -0.006824 | -0.0917734 | 0.02167076 | -2.274896 | 0.067389 | FALSE |
| Gross motor | Dyslexia | Indirect - Maternal | -0.00293 | 0.0348505 | -0.0407096 | 0.01927554 | -0.151983 | 0.879201 | FALSE |
| Gross motor | Dyslexia | Indirect - Paternal | 0.010913 | 0.0491386 | -0.027313 | 0.01950296 | 0.559548 | 0.702723 | FALSE |
| Language | Autism | Direct | 0.034338 | 0.0512153 | 0.01746151 | 0.00861067 | 3.987892 | 0.000836 | TRUE |
| Language | Autism | Indirect - Paternal | -0.01501 | -0.0005977 | -0.0294322 | 0.00735575 | -2.041253 | 0.128864 | FALSE |
| Language | Autism | Indirect - Maternal | -0.01158 | 0.0033628 | -0.0265245 | 0.0076243 | -1.51894 | 0.279979 | FALSE |
| Language | ADHD | Direct | 0.000661 | 0.0173403 | -0.0160178 | 0.00850973 | 0.077704 | 0.957208 | FALSE |
| Language | ADHD | Indirect - Paternal | 0.010276 | 0.0247388 | -0.0041871 | 0.00737905 | 1.392568 | 0.370832 | FALSE |
| Language | ADHD | Indirect - Maternal | 0.011861 | 0.026681 | -0.0029584 | 0.00756109 | 1.568727 | 0.265283 | FALSE |
| Language | EA | Direct | -0.02229 | -0.0047197 | -0.0398516 | 0.00896221 | -2.486625 | 0.043009 | TRUE |
| Language | EA | Indirect - Paternal | -0.01731 | -0.0024397 | -0.03218 | 0.00758682 | -2.281571 | 0.100823 | FALSE |
| Language | EA | Indirect - Maternal | -0.01838 | -0.0029811 | -0.0337765 | 0.00785597 | -2.339468 | 0.074305 | FALSE |
| Language | Cognitive Ability | Direct | -0.02953 | -0.0125928 | -0.0464734 | 0.00864302 | -3.416989 | 0.003964 | TRUE |
| Language | Cognitive Ability | Indirect - Paternal | -0.01893 | -0.0043199 | -0.0335497 | 0.00745657 | -2.53934 | 0.069452 | FALSE |
| Language | Cognitive Ability | Indirect - Maternal | -0.01355 | 0.0013793 | -0.028476 | 0.00761614 | -1.778903 | 0.209078 | FALSE |
| Language | Dyslexia | Direct | 0.016901 | 0.0332399 | 0.00056119 | 0.00833641 | 2.027318 | 0.101525 | FALSE |
| Language | Dyslexia | Indirect - Maternal | -0.00135 | 0.0132188 | -0.0159181 | 0.00743289 | -0.181579 | 0.873382 | FALSE |
| Language | Dyslexia | Indirect - Paternal | 0.006602 | 0.0210395 | -0.007835 | 0.00736594 | 0.896326 | 0.578262 | FALSE |
| Receptive language | Autism | Direct | 0.131693 | 0.2064952 | 0.05689014 | 0.03816454 | 3.450654 | 0.003964 | TRUE |
| Receptive language | Autism | Indirect - Paternal | -0.0431 | 0.0206902 | -0.1068872 | 0.03254526 | -1.324263 | 0.370832 | FALSE |
| Receptive language | Autism | Indirect - Maternal | -0.09052 | -0.0259081 | -0.1551245 | 0.03296337 | -2.745966 | 0.033415 | TRUE |
| Receptive language | ADHD | Direct | -0.01201 | 0.063402 | -0.0874199 | 0.03847498 | -0.312124 | 0.889133 | FALSE |
| Receptive language | ADHD | Indirect - Paternal | 0.009596 | 0.074042 | -0.0548496 | 0.03288052 | 0.29185 | 0.895815 | FALSE |
| Receptive language | ADHD | Indirect - Maternal | -0.01633 | 0.0502543 | -0.0829078 | 0.03396993 | -0.480624 | 0.769249 | FALSE |
| Receptive language | EA | Direct | -0.01908 | 0.0579044 | -0.0960684 | 0.03927877 | -0.485809 | 0.803978 | FALSE |
| Receptive language | EA | Indirect - Paternal | -0.00181 | 0.0653665 | -0.0689817 | 0.03427249 | -0.052742 | 0.977487 | FALSE |
| Receptive language | EA | Indirect - Maternal | -0.03883 | 0.0287341 | -0.106399 | 0.03447272 | -1.12647 | 0.448018 | FALSE |
| Receptive language | Cognitive Ability | Direct | -0.07105 | 0.0042594 | -0.1463544 | 0.03842189 | -1.849141 | 0.140081 | FALSE |
| Receptive language | Cognitive Ability | Indirect - Paternal | -0.00259 | 0.0633608 | -0.0685308 | 0.03364581 | -0.07683 | 0.977487 | FALSE |
| Receptive language | Cognitive Ability | Indirect - Maternal | -0.05308 | 0.011684 | -0.1178458 | 0.03304331 | -1.606404 | 0.265283 | FALSE |
| Receptive language | Dyslexia | Direct | 0.076592 | 0.1467987 | 0.00638623 | 0.0358195 | 2.138289 | 0.081233 | FALSE |
| Receptive language | Dyslexia | Indirect - Maternal | -0.02983 | 0.0322079 | -0.0918769 | 0.03165428 | -0.94251 | 0.508723 | FALSE |
| Receptive language | Dyslexia | Indirect - Paternal | -0.02096 | 0.0423676 | -0.0842802 | 0.0323081 | -0.648638 | 0.679086 | FALSE |
| Expressive language | Autism | Direct | 0.059993 | 0.096134 | 0.02385191 | 0.01843931 | 3.253536 | 0.006341 | TRUE |
| Expressive language | Autism | Indirect - Paternal | -0.03453 | -0.0031911 | -0.0658709 | 0.01598976 | -2.15957 | 0.110056 | FALSE |
| Expressive language | Autism | Indirect - Maternal | -0.0184 | 0.0134361 | -0.0502351 | 0.01624264 | -1.132788 | 0.448018 | FALSE |
| Expressive language | ADHD | Direct | 0.005556 | 0.0419282 | -0.0308168 | 0.0185574 | 0.299378 | 0.889133 | FALSE |
| Expressive language | ADHD | Indirect - Paternal | 0.015799 | 0.0472968 | -0.015699 | 0.01607034 | 0.98311 | 0.542605 | FALSE |
| Expressive language | ADHD | Indirect - Maternal | 0.034818 | 0.0666297 | 0.00300558 | 0.01623064 | 2.145179 | 0.099838 | FALSE |
| Expressive language | EA | Direct | -0.05516 | -0.0173655 | -0.0929461 | 0.01928078 | -2.860663 | 0.01763 | TRUE |
| Expressive language | EA | Indirect - Paternal | -0.04351 | -0.0111102 | -0.0759033 | 0.01652886 | -2.632171 | 0.060639 | FALSE |
| Expressive language | EA | Indirect - Maternal | -0.04522 | -0.0120118 | -0.0784234 | 0.01694175 | -2.669005 | 0.034603 | TRUE |
| Expressive language | Cognitive Ability | Direct | -0.06355 | -0.02716 | -0.0999448 | 0.01856754 | -3.422771 | 0.003964 | TRUE |
| Expressive language | Cognitive Ability | Indirect - Paternal | -0.04661 | -0.0148529 | -0.0783722 | 0.0162039 | -2.876628 | 0.033525 | TRUE |
| Expressive language | Cognitive Ability | Indirect - Maternal | -0.02607 | 0.0062106 | -0.0583508 | 0.01646975 | -1.582908 | 0.265283 | FALSE |
| Expressive language | Dyslexia | Direct | 0.019583 | 0.0550741 | -0.0159083 | 0.01810776 | 1.081464 | 0.49911 | FALSE |
| Expressive language | Dyslexia | Indirect - Maternal | 0.006801 | 0.0384823 | -0.0248794 | 0.01616368 | 0.420787 | 0.783621 | FALSE |
| Expressive language | Dyslexia | Indirect - Paternal | 0.020505 | 0.0518766 | -0.010867 | 0.01600601 | 1.28107 | 0.384522 | FALSE |
| RRBI | Autism | Direct | 0.002969 | 0.0204212 | -0.0144834 | 0.00890425 | 0.333423 | 0.889133 | FALSE |
| RRBI | Autism | Indirect - Paternal | 0.004938 | 0.0203359 | -0.0104607 | 0.00785629 | 0.628493 | 0.679086 | FALSE |
| RRBI | Autism | Indirect - Maternal | -0.00483 | 0.0106982 | -0.0203598 | 0.00792298 | -0.60972 | 0.706383 | FALSE |
| RRBI | ADHD | Direct | 0.007955 | 0.0254806 | -0.0095706 | 0.00894163 | 0.889661 | 0.583838 | FALSE |
| RRBI | ADHD | Indirect - Paternal | 0.012892 | 0.0282483 | -0.0024645 | 0.0078349 | 1.645442 | 0.249729 | FALSE |
| RRBI | ADHD | Indirect - Maternal | 0.010358 | 0.0255887 | -0.0048728 | 0.00777079 | 1.332932 | 0.361358 | FALSE |
| RRBI | EA | Direct | -0.00169 | 0.0161215 | -0.0194964 | 0.0090862 | -0.185718 | 0.947408 | FALSE |
| RRBI | EA | Indirect - Paternal | -0.01937 | -0.0037675 | -0.0349817 | 0.00796281 | -2.433133 | 0.074879 | FALSE |
| RRBI | EA | Indirect - Maternal | -0.01764 | -0.0018473 | -0.0334233 | 0.00805509 | -2.189337 | 0.095273 | FALSE |
| RRBI | Cognitive Ability | Direct | -0.00114 | 0.0164767 | -0.0187606 | 0.00898911 | -0.127039 | 0.956288 | FALSE |
| RRBI | Cognitive Ability | Indirect - Paternal | -0.01437 | 0.0012439 | -0.0299857 | 0.00796672 | -1.803868 | 0.209601 | FALSE |
| RRBI | Cognitive Ability | Indirect - Maternal | -0.02138 | -0.0059309 | -0.0368279 | 0.00788189 | -2.712469 | 0.033415 | TRUE |
| RRBI | Dyslexia | Direct | -0.00171 | 0.0154094 | -0.0188242 | 0.00873308 | -0.19551 | 0.947408 | FALSE |
| RRBI | Dyslexia | Indirect - Maternal | 0.004653 | 0.019946 | -0.0106405 | 0.0078027 | 0.596302 | 0.706383 | FALSE |
| RRBI | Dyslexia | Indirect - Paternal | 0.009833 | 0.0253874 | -0.0057212 | 0.00793588 | 1.239067 | 0.384522 | FALSE |
| Social & communication | Autism | Direct | 0.023405 | 0.0407 | 0.00610954 | 0.00882411 | 2.652369 | 0.028564 | TRUE |
| Social & communication | Autism | Indirect - Paternal | 0.005753 | 0.0210071 | -0.0095008 | 0.00778263 | 0.739226 | 0.676143 | FALSE |
| Social & communication | Autism | Indirect - Maternal | -0.00227 | 0.0130706 | -0.0176021 | 0.00782467 | -0.289566 | 0.839294 | FALSE |
| Social & communication | ADHD | Direct | 0.005494 | 0.0226366 | -0.0116486 | 0.00874623 | 0.62816 | 0.764174 | FALSE |
| Social & communication | ADHD | Indirect - Paternal | 0.007876 | 0.0227023 | -0.0069496 | 0.00756427 | 1.041254 | 0.513393 | FALSE |
| Social & communication | ADHD | Indirect - Maternal | -0.00276 | 0.012703 | -0.0182194 | 0.00788836 | -0.349656 | 0.807333 | FALSE |
| Social & communication | EA | Direct | 0.008342 | 0.0263066 | -0.0096233 | 0.0091658 | 0.910086 | 0.583838 | FALSE |
| Social & communication | EA | Indirect - Paternal | -0.01755 | -0.0020779 | -0.0330297 | 0.00789588 | -2.22316 | 0.100823 | FALSE |
| Social & communication | EA | Indirect - Maternal | -0.0209 | -0.005039 | -0.0367524 | 0.00809015 | -2.58286 | 0.040851 | TRUE |
| Social & communication | Cognitive Ability | Direct | 0.005422 | 0.0225481 | -0.0117042 | 0.00873783 | 0.620519 | 0.764174 | FALSE |
| Social & communication | Cognitive Ability | Indirect - Paternal | -0.01713 | -0.002132 | -0.0321377 | 0.00765453 | -2.238527 | 0.100823 | FALSE |
| Social & communication | Cognitive Ability | Indirect - Maternal | -0.00298 | 0.012217 | -0.0181742 | 0.00775286 | -0.384188 | 0.796412 | FALSE |
| Social & communication | Dyslexia | Direct | -0.00426 | 0.0127056 | -0.0212268 | 0.00865621 | -0.492201 | 0.803978 | FALSE |
| Social & communication | Dyslexia | Indirect - Maternal | -0.00347 | 0.0115401 | -0.0184874 | 0.00766008 | -0.45348 | 0.774056 | FALSE |
| Social & communication | Dyslexia | Indirect - Paternal | 0.004841 | 0.0198867 | -0.0102042 | 0.00767626 | 0.63068 | 0.679086 | FALSE |

p.value.adj – adjusted p value correction (FDR); sig after MT – True/False value if statistically significant after multiple testing corrections

Supplementary Table 6: Variance explained for direct vs. indirect effect in multi-trait trio-PGS models.

| Scale | % variance explained direct | % variance explained indirect | ratio direct:indirect | % variance explained direct no sibs | % variance explained indirect no sibs | ratio direct:indirect no sibs |
| --- | --- | --- | --- | --- | --- | --- |
| Social & communication (SCQ) | 0.03 | 0.07 | 0.45 | 0.04 | 0.07 | 0.54 |
| RRBI (SCQ) | 0 | 0.06 | 0.06 | 0.01 | 0.07 | 0.17 |
| Attention (CBCL) | 0.27 | 0.09 | 3.02 | 0.25 | 0.08 | 3.01 |
| Hyperactivity (CBCL) | 0.06 | 0.12 | 0.51 | 0.07 | 0.13 | 0.54 |
| Motor (ASQ) | 0.05 | 0.23 | 0.20 | 0.05 | 0.20 | 0.27 |
| Language (ASQ) | 0.16 | 0.07 | 2.24 | 0.16 | 0.08 | 2.02 |

No sibs – models only include one sibling per family

# **Results for post-hoc analyses using SCQ total score:**

Trio-GCTA and trio-PGS models were also run for a total score of the SCQ. Results indicated the influence of both direct and indirect effects. For the trio-PGS analyses, maternal and paternal indirect effects were found for the EA and cognitive ability (negatively associated with higher total SCQ scores) with no corrections for multiple testing. These effects were almost all present for the RRBI and social communication subscales however, only the effect of maternal PGS for cognitive ability on RRBI and maternal PGS for educational attainment on social communication remained significant after multiple testing corrections in the main analysis. A paternal indirect effect of the ADHD PGS was also found for the total SCQ score, but this just reached statistical significance (*β* = 0.02 [0.01 – 0.03], uncorrected p = 0.039). No direct effects were found.

The best fitting model for trio-GCTA was inconclusive as all models except the null model had almost identical AIC values. Regardless of the model, direct effects were estimated between 3.7% (no covariance model) and 5.2% (direct effects only model) which is similar to the direct effects estimated for the social communication subscale (5.7% in the direct effects only model). For the two models estimating indirect effects, maternal and paternal effects jointly accounted for 4.1% (no covariance model) - 7.1% (full model), with maternal and paternal effects being equal in the full model and primarily maternal indirect effects when not modeling covariances. These estimates for indirect effects are similar but slightly smaller in magnitude to the findings for the RRBI subscale.

Supplementary table 7: trio-GCTA model fit indices for SCQ total score

| Model | −2ll | AIC | BIC | df | Δdf | | *p*-value |
| --- | --- | --- | --- | --- | --- | --- | --- |
| SCQ total score |  |  |  |  |  |  | |
| Full | 45720.6 | 45804.6 | 46128.57 | 42 |  |  | |
| No covariance | 45726.91 | 45804.91 | 46105.74 | 39 | -3 | 0.097 | |
| Direct only | 45730.65 | 45804.65 | 46090.05 | 37 | -2 | 0.15 | |
| Null | 45737.81 | 45809.81 | 46087.5 | 36 | -1 | 0.007 | |

Supplementary table 8: Model parameters for the single PGS-trait model for SCQ total score

| **Predictor** | **Effect** | **Estimate** | **higher_ci** | **lower_ci** | **std.error** | **p.value** |
| --- | --- | --- | --- | --- | --- | --- |
| autism | Direct | 0.015151 | 0.032311 | -0.00201 | 0.008755 | 0.083529 |
| autism | Indirect - Paternal | 0.006411 | 0.02148 | -0.00866 | 0.007688 | 0.404313 |
| autism | Indirect - Maternal | -0.00552 | 0.00976 | -0.02081 | 0.007797 | 0.478779 |
| ADHD | Direct | 0.006894 | 0.024012 | -0.01022 | 0.008734 | 0.429917 |
| ADHD | Indirect - Paternal | 0.015646 | 0.030501 | 0.00079 | 0.00758 | 0.039009 |
| ADHD | Indirect - Maternal | 0.008306 | 0.023411 | -0.0068 | 0.007707 | 0.281134 |
| EA | Direct | 0.004606 | 0.022283 | -0.01307 | 0.009019 | 0.609521 |
| EA | Indirect - Paternal | -0.02729 | -0.01202 | -0.04256 | 0.007791 | 0.000462 |
| EA | Indirect - Maternal | -0.02746 | -0.01196 | -0.04296 | 0.007908 | 0.000516 |
| cog | Direct | 0.001886 | 0.019082 | -0.01531 | 0.008774 | 0.829811 |
| cog | Indirect - Paternal | -0.02176 | -0.00666 | -0.03687 | 0.007706 | 0.004746 |
| cog | Indirect - Maternal | -0.02033 | -0.00512 | -0.03554 | 0.007759 | 0.008793 |
| dyslexia | Direct | -0.00199 | 0.014681 | -0.01866 | 0.008506 | 0.814927 |
| dyslexia | Indirect - Maternal | 0.000101 | 0.015002 | -0.0148 | 0.007603 | 0.989386 |
| dyslexia | Indirect - Paternal | 0.00992 | 0.024998 | -0.00516 | 0.007692 | 0.197194 |

Rows of statistically significant PGS effects highlighted. No multiple testing corrections applied to p values.

Supplementary table 9: trio-GCTA model variance component estimates for SCQ total score

| model | o | m | p | om | op | mp | e |
| --- | --- | --- | --- | --- | --- | --- | --- |
| full | 0.041 | 0.034 | 0.037 | 0.01 | -0.025 | 0.02 | 0.904 |
| nocov | 0.037 | 0.038 | 0.003 | 0 | 0 | 0 | 0.922 |
| direct | 0.052 | 0 | 0 | 0 | 0 | 0 | 0.948 |
| null | 0 | 0 | 0 | 0 | 0 | 0 | 1 |

o – variance offspring. m -variance mother. p – variance father. om -covariance between child and mother . op – covariance between child and father. mp – covariance between mother and father. e – residual variance

# **Original Results using PGS created in PRSice2 using the pre-registered method**

Supplementary Figure 3: Direct and Indirect Genetic Effects of ADHD and autism PGS on Early Neurodevelopmental Traits using PGS created with PRScise2


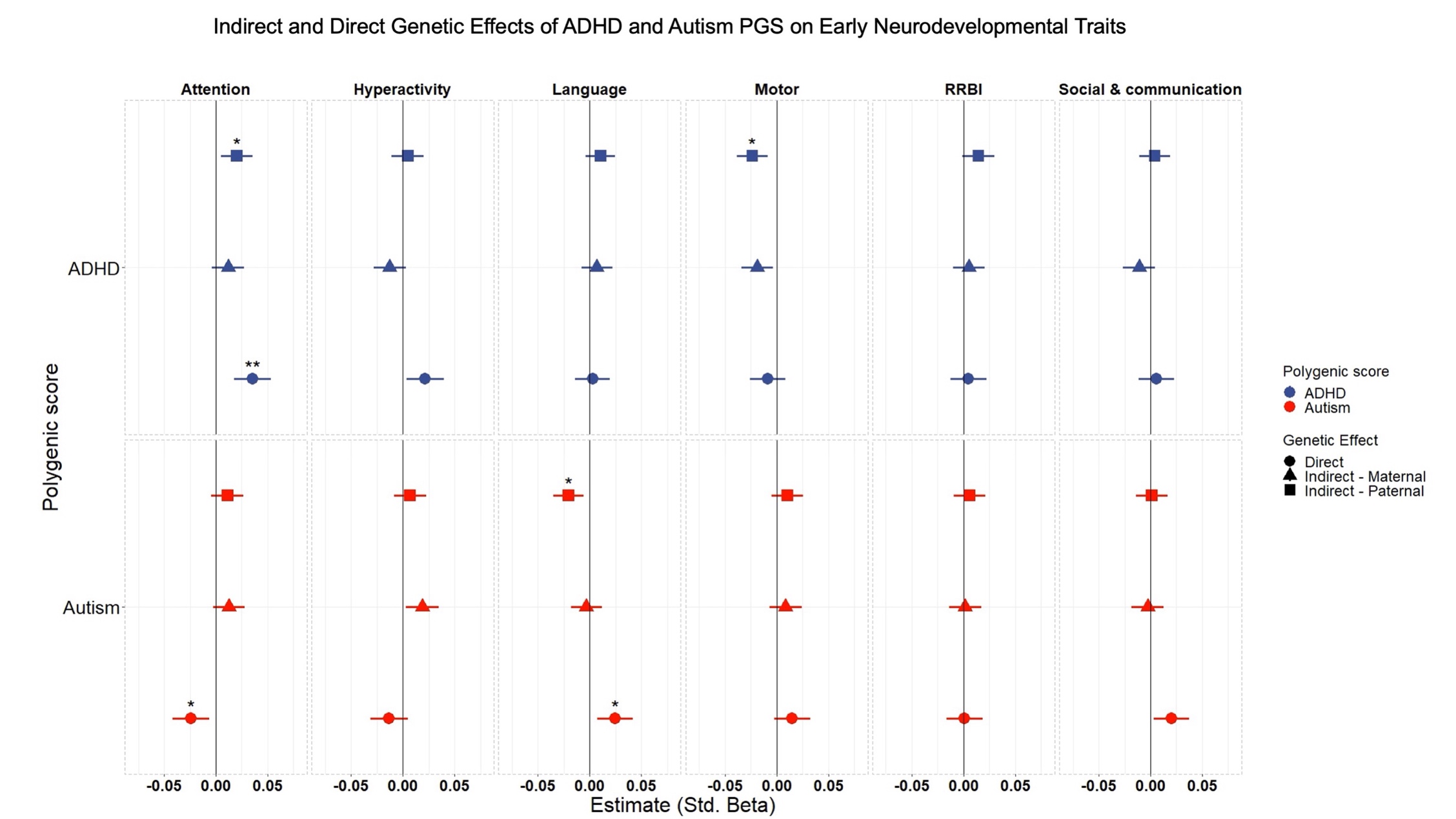


Figure S3: Standardized beta estimates for ADHD and autism PGS on the six measures of early neurodevelopmental traits. 95% confidence intervals are shown. “*”, “**”, “***” denote adjusted p-values <0.05, <0.01, and <0.001 after multiple testing correction. All results presented are the PGS effect adjusting for the effect of the PGS for the other members of the trio.


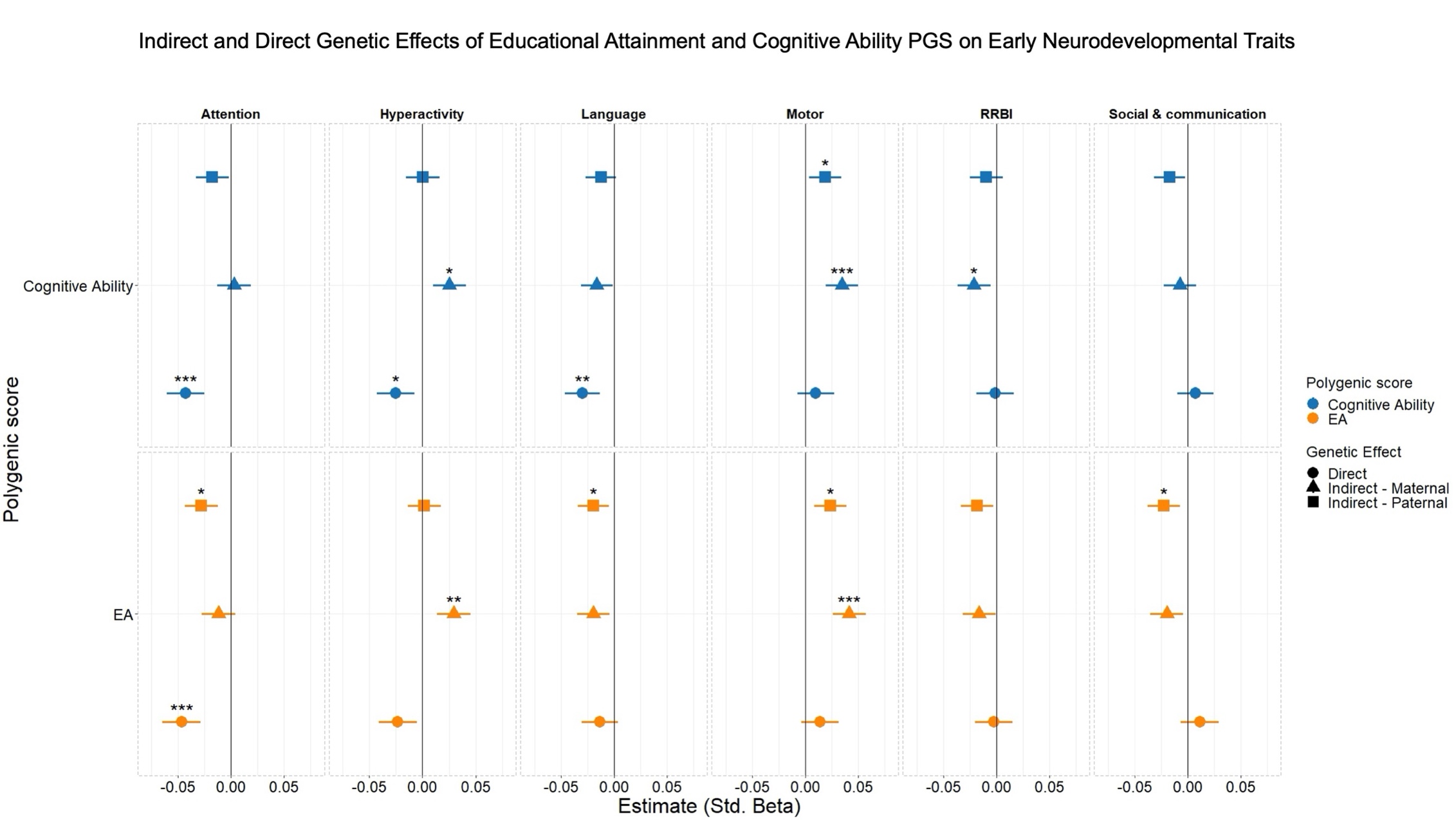
Supplementary Figure 4: Direct and Indirect Genetic Effects of Educational Attainment and Cognitive Ability PGS on Early Neurodevelopmental Traits using PGS created with PRScise2

Figure 4 Standardized beta estimates for EA and cognitive ability PGS on the six measures of early neurodevelopmental traits. 95% confidence intervals are shown. “*”, “**”, “***” denote adjusted p-values <0.05, <0.01, and <0.001 after multiple testing correction. All results presented are the PGS effect adjusting for the effect of the PGS for the other members of the trio.


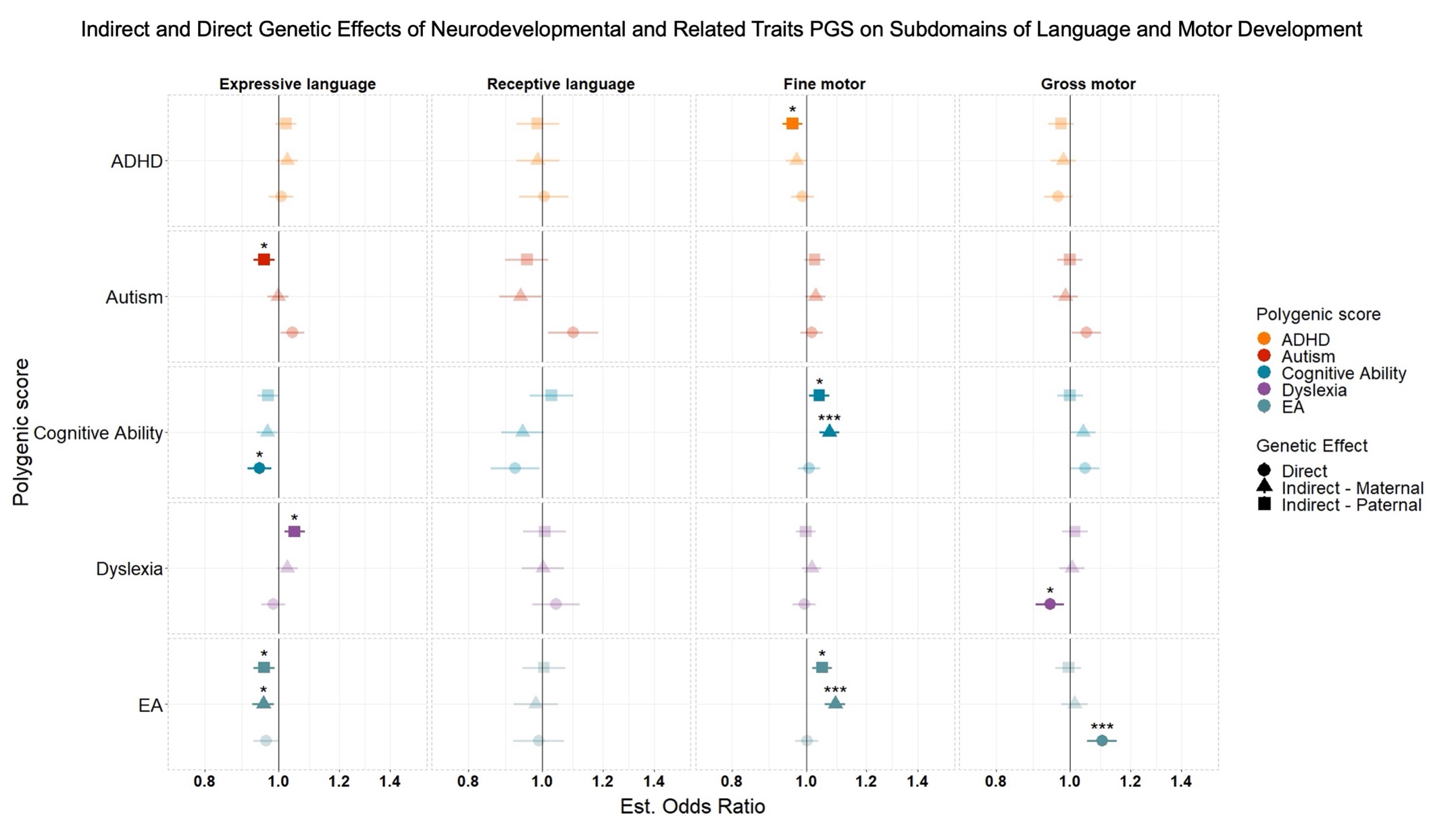
Supplementary Figure 5: Direct and Indirect Genetic Effects of PGS on subdomains of Language and Motor Development using PGS created with PRScise2

Figure S5: estimated log odds ratio for all PGS on likelihood of reporting difficulties in the subdomains of language and motor difficulties. 95% confidence intervals are shown. “*”, “**”, “***” denote adjusted p-values <0.05, <0.01, and <0.001 after multiple testing

Supplementary Table 10: Model parameters for the single PGS-trait models using PGS created with PRScise2

| **outcome** | **predictor** | **effect** | **Estimate** | **higher_ci** | **lower_ci** | **std.error** | **z or t value** | **p.value.adj** | **sig after MT** |
| --- | --- | --- | --- | --- | --- | --- | --- | --- | --- |
| Inattention | Autism | Direct | -0.02408 | -0.00623 | -0.04193 | 0.009107 | -2.6444 | 0.04094448 | TRUE |
| Inattention | Autism | Indirect - Paternal | 0.011376 | 0.026861 | -0.00411 | 0.0079 | 1.440006 | 0.30219979 | FALSE |
| Inattention | Autism | Indirect - Maternal | 0.012693 | 0.027996 | -0.00261 | 0.007807 | 1.625812 | 0.24762489 | FALSE |
| Inattention | ADHD | Direct | 0.035704 | 0.053524 | 0.017883 | 0.009092 | 3.926863 | 0.0010787 | TRUE |
| Inattention | ADHD | Indirect - Paternal | 0.020054 | 0.035313 | 0.004795 | 0.007785 | 2.575836 | 0.04130243 | TRUE |
| Inattention | ADHD | Indirect - Maternal | 0.011875 | 0.027523 | -0.00377 | 0.007984 | 1.487395 | 0.26880809 | FALSE |
| Inattention | EA | Direct | -0.04661 | -0.02864 | -0.06458 | 0.009168 | -5.08358 | 1.8655E-05 | TRUE |
| Inattention | EA | Indirect - Paternal | -0.02812 | -0.01253 | -0.04372 | 0.007957 | -3.53428 | 0.02048172 | TRUE |
| Inattention | EA | Indirect - Maternal | -0.01185 | 0.003878 | -0.02757 | 0.008023 | -1.47666 | 0.26880809 | FALSE |
| Inattention | Cog. Ability | Direct | -0.04303 | -0.0253 | -0.06077 | 0.009048 | -4.75612 | 4.9619E-05 | TRUE |
| Inattention | Cog. Ability | Indirect - Paternal | -0.0177 | -0.00213 | -0.03327 | 0.007945 | -2.22759 | 0.07199135 | FALSE |
| Inattention | Cog. Ability | Indirect - Maternal | 0.002693 | 0.018482 | -0.0131 | 0.008056 | 0.334247 | 0.81611141 | FALSE |
| Inattention | Dyslexia | Direct | 0.008479 | 0.025804 | -0.00885 | 0.00884 | 0.959161 | 0.53688814 | FALSE |
| Inattention | Dyslexia | Indirect - Maternal | -0.00356 | 0.011753 | -0.01887 | 0.007812 | -0.45547 | 0.79003972 | FALSE |
| Inattention | Dyslexia | Indirect - Paternal | 0.002824 | 0.018154 | -0.01251 | 0.007821 | 0.361062 | 0.89756984 | FALSE |
| Hyperactivity | Autism | Direct | -0.0132 | 0.0047 | -0.0311 | 0.009131 | -1.44534 | 0.30721295 | FALSE |
| Hyperactivity | Autism | Indirect - Paternal | 0.006977 | 0.022505 | -0.00855 | 0.007923 | 0.880607 | 0.59146717 | FALSE |
| Hyperactivity | Autism | Indirect - Maternal | 0.018972 | 0.034829 | 0.003115 | 0.00809 | 2.345007 | 0.07931093 | FALSE |
| Hyperactivity | ADHD | Direct | 0.02162 | 0.039463 | 0.003777 | 0.009104 | 2.374931 | 0.06753795 | FALSE |
| Hyperactivity | ADHD | Indirect - Paternal | 0.004717 | 0.020198 | -0.01076 | 0.007898 | 0.59727 | 0.76435052 | FALSE |
| Hyperactivity | ADHD | Indirect - Maternal | -0.01257 | 0.003072 | -0.02822 | 0.007982 | -1.57509 | 0.25340211 | FALSE |
| Hyperactivity | EA | Direct | -0.02321 | -0.00508 | -0.04134 | 0.00925 | -2.50941 | 0.05499852 | FALSE |
| Hyperactivity | EA | Indirect - Paternal | 0.001762 | 0.017441 | -0.01392 | 0.008 | 0.220209 | 0.95701875 | FALSE |
| Hyperactivity | EA | Indirect - Maternal | 0.029632 | 0.045449 | 0.013815 | 0.00807 | 3.672009 | 0.00241162 | TRUE |
| Hyperactivity | Cog. Ability | Direct | -0.02555 | -0.00783 | -0.04326 | 0.009039 | -2.82629 | 0.03238696 | TRUE |
| Hyperactivity | Cog. Ability | Indirect - Paternal | 0.000456 | 0.016292 | -0.01538 | 0.008079 | 0.056487 | 0.98355681 | FALSE |
| Hyperactivity | Cog. Ability | Indirect - Maternal | 0.02553 | 0.041253 | 0.009807 | 0.008022 | 3.182541 | 0.01218088 | TRUE |
| Hyperactivity | Dyslexia | Direct | 0.001131 | 0.018698 | -0.01644 | 0.008963 | 0.126129 | 0.93822607 | FALSE |
| Hyperactivity | Dyslexia | Indirect - Maternal | -0.00924 | 0.006297 | -0.02478 | 0.007927 | -1.16559 | 0.42974231 | FALSE |
| Hyperactivity | Dyslexia | Indirect - Paternal | -0.00206 | 0.013496 | -0.01761 | 0.007935 | -0.2591 | 0.95701875 | FALSE |
| Motor | Autism | Direct | 0.014966 | 0.032333 | -0.0024 | 0.008861 | 1.689049 | 0.22805709 | FALSE |
| Motor | Autism | Indirect - Paternal | 0.010519 | 0.025811 | -0.00477 | 0.007802 | 1.348205 | 0.30621524 | FALSE |
| Motor | Autism | Indirect - Maternal | 0.008741 | 0.024014 | -0.00653 | 0.007792 | 1.121702 | 0.4366672 | FALSE |
| Motor | ADHD | Direct | -0.00876 | 0.008477 | -0.026 | 0.008794 | -0.99605 | 0.53688814 | FALSE |
| Motor | ADHD | Indirect - Paternal | -0.02351 | -0.00871 | -0.03831 | 0.007552 | -3.11345 | 0.02559064 | TRUE |
| Motor | ADHD | Indirect - Maternal | -0.01876 | -0.00351 | -0.03401 | 0.00778 | -2.41115 | 0.07231592 | FALSE |
| Motor | EA | Direct | 0.014261 | 0.031823 | -0.0033 | 0.00896 | 1.591593 | 0.25391026 | FALSE |
| Motor | EA | Indirect - Paternal | 0.023769 | 0.038982 | 0.008557 | 0.007762 | 3.062449 | 0.02559064 | TRUE |
| Motor | EA | Indirect - Maternal | 0.041771 | 0.057425 | 0.026118 | 0.007987 | 5.230223 | 4.2674E-06 | TRUE |
| Motor | Cog. Ability | Direct | 0.01023 | 0.027387 | -0.00693 | 0.008754 | 1.16866 | 0.4491702 | FALSE |
| Motor | Cog. Ability | Indirect - Paternal | 0.018975 | 0.034141 | 0.003808 | 0.007738 | 2.452169 | 0.04735573 | TRUE |
| Motor | Cog. Ability | Indirect - Maternal | 0.034804 | 0.050061 | 0.019546 | 0.007784 | 4.47102 | 9.7745E-05 | TRUE |
| Motor | Dyslexia | Direct | -0.01252 | 0.004675 | -0.02971 | 0.008771 | -1.42695 | 0.30721295 | FALSE |
| Motor | Dyslexia | Indirect - Maternal | 0.007864 | 0.023022 | -0.00729 | 0.007734 | 1.016825 | 0.48319763 | FALSE |
| Motor | Dyslexia | Indirect - Paternal | 0.004241 | 0.01952 | -0.01104 | 0.007795 | 0.544071 | 0.77524484 | FALSE |
| Fine motor | Autism | Direct | 0.016965 | 0.051276 | -0.01735 | 0.017506 | 0.969109 | 0.53688814 | FALSE |
| Fine motor | Autism | Indirect - Paternal | 0.025526 | 0.055888 | -0.00484 | 0.015491 | 1.647811 | 0.21609649 | FALSE |
| Fine motor | Autism | Indirect - Maternal | 0.027535 | 0.05775 | -0.00268 | 0.015416 | 1.786141 | 0.18522217 | FALSE |
| Fine motor | ADHD | Direct | -0.01086 | 0.023392 | -0.04512 | 0.017477 | -0.62159 | 0.74197009 | FALSE |
| Fine motor | ADHD | Indirect - Paternal | -0.04094 | -0.01136 | -0.07052 | 0.015094 | -2.71236 | 0.03612798 | TRUE |
| Fine motor | ADHD | Indirect - Maternal | -0.02976 | 0.000527 | -0.06004 | 0.015451 | -1.92588 | 0.15036265 | FALSE |
| Fine motor | EA | Direct | 0.001808 | 0.036845 | -0.03323 | 0.017876 | 0.101113 | 0.93822607 | FALSE |
| Fine motor | EA | Indirect - Paternal | 0.048277 | 0.078563 | 0.01799 | 0.015452 | 3.124274 | 0.02559064 | TRUE |
| Fine motor | EA | Indirect - Maternal | 0.087304 | 0.11845 | 0.056158 | 0.015891 | 5.493982 | 1.9844E-06 | TRUE |
| Fine motor | Cog. Ability | Direct | 0.008755 | 0.042963 | -0.02545 | 0.017453 | 0.501635 | 0.81043287 | FALSE |
| Fine motor | Cog. Ability | Indirect - Paternal | 0.039523 | 0.069704 | 0.009341 | 0.015399 | 2.566636 | 0.04130243 | TRUE |
| Fine motor | Cog. Ability | Indirect - Maternal | 0.070187 | 0.100561 | 0.039812 | 0.015497 | 4.528991 | 9.7745E-05 | TRUE |
| Fine motor | Dyslexia | Direct | -0.0062 | 0.02819 | -0.0406 | 0.017548 | -0.35359 | 0.88010333 | FALSE |
| Fine motor | Dyslexia | Indirect - Maternal | 0.017544 | 0.047387 | -0.0123 | 0.015226 | 1.152199 | 0.42974231 | FALSE |
| Fine motor | Dyslexia | Indirect - Paternal | -0.00133 | 0.029129 | -0.03178 | 0.015538 | -0.08537 | 0.98355681 | FALSE |
| Gross motor | Autism | Direct | 0.049617 | 0.092831 | 0.006404 | 0.022048 | 2.250443 | 0.07942139 | FALSE |
| Gross motor | Autism | Indirect - Paternal | -0.0004 | 0.037569 | -0.03837 | 0.019371 | -0.02061 | 0.98355681 | FALSE |
| Gross motor | Autism | Indirect - Maternal | -0.01426 | 0.023607 | -0.05213 | 0.019321 | -0.73814 | 0.63948273 | FALSE |
| Gross motor | ADHD | Direct | -0.03507 | 0.008147 | -0.07829 | 0.022049 | -1.59051 | 0.25391026 | FALSE |
| Gross motor | ADHD | Indirect - Paternal | -0.02662 | 0.011337 | -0.06458 | 0.019368 | -1.37467 | 0.30621524 | FALSE |
| Gross motor | ADHD | Indirect - Maternal | -0.01991 | 0.018268 | -0.0581 | 0.01948 | -1.02226 | 0.48319763 | FALSE |
| Gross motor | EA | Direct | 0.096856 | 0.141413 | 0.052299 | 0.022733 | 4.260583 | 0.00033982 | TRUE |
| Gross motor | EA | Indirect - Paternal | -0.00426 | 0.034586 | -0.04311 | 0.01982 | -0.21503 | 0.95701875 | FALSE |
| Gross motor | EA | Indirect - Maternal | 0.014101 | 0.053615 | -0.02541 | 0.02016 | 0.699462 | 0.65440952 | FALSE |
| Gross motor | Cog. Ability | Direct | 0.045483 | 0.088761 | 0.002204 | 0.022081 | 2.059809 | 0.10949123 | FALSE |
| Gross motor | Cog. Ability | Indirect - Paternal | 0.000821 | 0.039447 | -0.03781 | 0.019707 | 0.041654 | 0.98355681 | FALSE |
| Gross motor | Cog. Ability | Indirect - Maternal | 0.038876 | 0.077442 | 0.00031 | 0.019677 | 1.975767 | 0.14170947 | FALSE |
| Gross motor | Dyslexia | Direct | -0.06033 | -0.01744 | -0.10321 | 0.021879 | -2.75721 | 0.03238696 | TRUE |
| Gross motor | Dyslexia | Indirect - Maternal | 0.006134 | 0.043996 | -0.03173 | 0.019317 | 0.317555 | 0.81611141 | FALSE |
| Gross motor | Dyslexia | Indirect - Paternal | 0.015044 | 0.053213 | -0.02313 | 0.019474 | 0.772498 | 0.63174453 | FALSE |
| Language | Autism | Direct | 0.024232 | 0.041269 | 0.007194 | 0.008693 | 2.787597 | 0.03238696 | TRUE |
| Language | Autism | Indirect - Paternal | -0.02039 | -0.00572 | -0.03506 | 0.007485 | -2.72396 | 0.03612798 | TRUE |
| Language | Autism | Indirect - Maternal | -0.00313 | 0.011701 | -0.01795 | 0.007565 | -0.41324 | 0.79003972 | FALSE |
| Language | ADHD | Direct | 0.002855 | 0.019668 | -0.01396 | 0.008578 | 0.332802 | 0.88010333 | FALSE |
| Language | ADHD | Indirect - Paternal | 0.010277 | 0.024707 | -0.00415 | 0.007362 | 1.395901 | 0.30621524 | FALSE |
| Language | ADHD | Indirect - Maternal | 0.006785 | 0.021611 | -0.00804 | 0.007565 | 0.896907 | 0.54122032 | FALSE |
| Language | EA | Direct | -0.01314 | 0.004095 | -0.03038 | 0.008795 | -1.49434 | 0.29369213 | FALSE |
| Language | EA | Indirect - Paternal | -0.01919 | -0.00445 | -0.03394 | 0.007523 | -2.55129 | 0.04130243 | TRUE |
| Language | EA | Indirect - Maternal | -0.0194 | -0.00416 | -0.03464 | 0.007773 | -2.49579 | 0.06985654 | FALSE |
| Language | Cog. Ability | Direct | -0.02966 | -0.01295 | -0.04636 | 0.008522 | -3.48018 | 0.00501949 | TRUE |
| Language | Cog. Ability | Indirect - Paternal | -0.01211 | 0.00224 | -0.02646 | 0.007321 | -1.65406 | 0.21609649 | FALSE |
| Language | Cog. Ability | Indirect - Maternal | -0.01605 | -0.00103 | -0.03106 | 0.007662 | -2.09439 | 0.13712226 | FALSE |
| Language | Dyslexia | Direct | -0.00025 | 0.016117 | -0.01662 | 0.008353 | -0.03038 | 0.97576225 | FALSE |
| Language | Dyslexia | Indirect - Maternal | 0.006523 | 0.021052 | -0.00801 | 0.007412 | 0.880025 | 0.54122032 | FALSE |
| Language | Dyslexia | Indirect - Paternal | 0.018527 | 0.032978 | 0.004076 | 0.007373 | 2.512858 | 0.04279319 | TRUE |
| Receptive language | Autism | Direct | 0.093126 | 0.168457 | 0.017795 | 0.038434 | 2.42301 | 0.06413549 | FALSE |
| Receptive language | Autism | Indirect - Paternal | -0.04695 | 0.017149 | -0.11106 | 0.032706 | -1.43566 | 0.30219979 | FALSE |
| Receptive language | Autism | Indirect - Maternal | -0.06655 | -0.00253 | -0.13056 | 0.032662 | -2.03745 | 0.13712226 | FALSE |
| Receptive language | ADHD | Direct | 0.004655 | 0.079194 | -0.06989 | 0.038031 | 0.12239 | 0.93822607 | FALSE |
| Receptive language | ADHD | Indirect - Paternal | -0.01412 | 0.050058 | -0.07829 | 0.032742 | -0.43112 | 0.85433289 | FALSE |
| Receptive language | ADHD | Indirect - Maternal | -0.01395 | 0.050613 | -0.07851 | 0.032939 | -0.42344 | 0.79003972 | FALSE |
| Receptive language | EA | Direct | -0.01105 | 0.065714 | -0.08781 | 0.039164 | -0.28208 | 0.90451839 | FALSE |
| Receptive language | EA | Indirect - Paternal | 0.004236 | 0.069647 | -0.06118 | 0.033373 | 0.126917 | 0.9771806 | FALSE |
| Receptive language | EA | Indirect - Maternal | -0.01992 | 0.046657 | -0.0865 | 0.033969 | -0.58646 | 0.69695512 | FALSE |
| Receptive language | Cog. Ability | Direct | -0.08199 | -0.00865 | -0.15532 | 0.037416 | -2.19121 | 0.08363764 | FALSE |
| Receptive language | Cog. Ability | Indirect - Paternal | 0.026916 | 0.092021 | -0.03819 | 0.033217 | 0.810309 | 0.63174453 | FALSE |
| Receptive language | Cog. Ability | Indirect - Maternal | -0.05988 | 0.004839 | -0.12461 | 0.033022 | -1.81345 | 0.18358522 | FALSE |
| Receptive language | Dyslexia | Direct | 0.040745 | 0.112409 | -0.03092 | 0.036563 | 1.114384 | 0.47341888 | FALSE |
| Receptive language | Dyslexia | Indirect - Maternal | 0.001397 | 0.064409 | -0.06162 | 0.032149 | 0.043448 | 0.98504569 | FALSE |
| Receptive language | Dyslexia | Indirect - Paternal | 0.006529 | 0.071177 | -0.05812 | 0.032984 | 0.19794 | 0.95701875 | FALSE |
| Expressive language | Autism | Direct | 0.04146 | 0.077815 | 0.005104 | 0.018549 | 2.235175 | 0.07942139 | FALSE |
| Expressive language | Autism | Indirect - Paternal | -0.04352 | -0.01194 | -0.07509 | 0.01611 | -2.70122 | 0.03612798 | TRUE |
| Expressive language | Autism | Indirect - Maternal | -0.00194 | 0.029749 | -0.03363 | 0.016169 | -0.12013 | 0.94206312 | FALSE |
| Expressive language | ADHD | Direct | 0.007262 | 0.043592 | -0.02907 | 0.018536 | 0.391756 | 0.88010333 | FALSE |
| Expressive language | ADHD | Indirect - Paternal | 0.021663 | 0.053059 | -0.00973 | 0.016019 | 1.352338 | 0.30621524 | FALSE |
| Expressive language | ADHD | Indirect - Maternal | 0.025387 | 0.057208 | -0.00643 | 0.016235 | 1.563749 | 0.25340211 | FALSE |
| Expressive language | EA | Direct | -0.03734 | -9.2E-05 | -0.07458 | 0.019003 | -1.96484 | 0.13011595 | FALSE |
| Expressive language | EA | Indirect - Paternal | -0.04387 | -0.01186 | -0.07587 | 0.016328 | -2.68649 | 0.03612798 | TRUE |
| Expressive language | EA | Indirect - Maternal | -0.04649 | -0.01357 | -0.0794 | 0.016792 | -2.76837 | 0.040271 | TRUE |
| Expressive language | Cog. Ability | Direct | -0.05718 | -0.02115 | -0.09321 | 0.018383 | -3.11076 | 0.01556854 | TRUE |
| Expressive language | Cog. Ability | Indirect - Paternal | -0.03178 | -0.00035 | -0.0632 | 0.016032 | -1.98206 | 0.11870918 | FALSE |
| Expressive language | Cog. Ability | Indirect - Maternal | -0.03339 | -0.00111 | -0.06567 | 0.016468 | -2.02735 | 0.13712226 | FALSE |
| Expressive language | Dyslexia | Direct | -0.01689 | 0.018854 | -0.05264 | 0.018238 | -0.92621 | 0.53688814 | FALSE |
| Expressive language | Dyslexia | Indirect - Maternal | 0.025059 | 0.056787 | -0.00667 | 0.016188 | 1.548009 | 0.25340211 | FALSE |
| Expressive language | Dyslexia | Indirect - Paternal | 0.048193 | 0.079506 | 0.01688 | 0.015976 | 3.016579 | 0.02559064 | TRUE |
| RRBI | Autism | Direct | 0.000908 | 0.018332 | -0.01652 | 0.00889 | 0.102148 | 0.93822607 | FALSE |
| RRBI | Autism | Indirect - Paternal | 0.006 | 0.021303 | -0.0093 | 0.007808 | 0.76846 | 0.63174453 | FALSE |
| RRBI | Autism | Indirect - Maternal | 0.001483 | 0.016938 | -0.01397 | 0.007885 | 0.188057 | 0.90514188 | FALSE |
| RRBI | ADHD | Direct | 0.00475 | 0.022263 | -0.01276 | 0.008935 | 0.531655 | 0.80401321 | FALSE |
| RRBI | ADHD | Indirect - Paternal | 0.014356 | 0.029596 | -0.00089 | 0.007776 | 1.846139 | 0.15448586 | FALSE |
| RRBI | ADHD | Indirect - Maternal | 0.005194 | 0.020451 | -0.01006 | 0.007784 | 0.667337 | 0.66389904 | FALSE |
| RRBI | EA | Direct | -0.00213 | 0.015479 | -0.01973 | 0.008982 | -0.23666 | 0.92377993 | FALSE |
| RRBI | EA | Indirect - Paternal | -0.01806 | -0.00266 | -0.03345 | 0.007853 | -2.29936 | 0.06716498 | FALSE |
| RRBI | EA | Indirect - Maternal | -0.01606 | -0.00044 | -0.03168 | 0.007969 | -2.01535 | 0.13712226 | FALSE |
| RRBI | Cog. Ability | Direct | -0.00099 | 0.016583 | -0.01856 | 0.008964 | -0.11009 | 0.93822607 | FALSE |
| RRBI | Cog. Ability | Indirect - Paternal | -0.0094 | 0.006057 | -0.02486 | 0.007888 | -1.19213 | 0.38870363 | FALSE |
| RRBI | Cog. Ability | Indirect - Maternal | -0.02099 | -0.00556 | -0.03641 | 0.007871 | -2.66625 | 0.04797083 | TRUE |
| RRBI | Dyslexia | Direct | 0.00821 | 0.02538 | -0.00896 | 0.00876 | 0.937148 | 0.53688814 | FALSE |
| RRBI | Dyslexia | Indirect - Maternal | 0.00013 | 0.01545 | -0.01519 | 0.007816 | 0.016606 | 0.986751 | FALSE |
| RRBI | Dyslexia | Indirect - Paternal | 0.007018 | 0.022359 | -0.00832 | 0.007827 | 0.896687 | 0.59146717 | FALSE |
| Social & communication | Autism | Direct | 0.020167 | 0.037466 | 0.002867 | 0.008826 | 2.284802 | 0.07942139 | FALSE |
| Social & communication | Autism | Indirect - Paternal | 0.001364 | 0.016672 | -0.01394 | 0.00781 | 0.1747 | 0.95701875 | FALSE |
| Social & communication | Autism | Indirect - Maternal | -0.00286 | 0.012406 | -0.01813 | 0.00779 | -0.36729 | 0.81068761 | FALSE |
| Social & communication | ADHD | Direct | 0.005615 | 0.022871 | -0.01164 | 0.008804 | 0.637839 | 0.74197009 | FALSE |
| Social & communication | ADHD | Indirect - Paternal | 0.00406 | 0.018797 | -0.01068 | 0.007519 | 0.540023 | 0.77524484 | FALSE |
| Social & communication | ADHD | Indirect - Maternal | -0.01105 | 0.004341 | -0.02645 | 0.007854 | -1.40724 | 0.29512674 | FALSE |
| Social & communication | EA | Direct | 0.011445 | 0.029231 | -0.00634 | 0.009074 | 1.261274 | 0.39850389 | FALSE |
| Social & communication | EA | Indirect - Paternal | -0.02257 | -0.00719 | -0.03794 | 0.007844 | -2.877 | 0.0334856 | TRUE |
| Social & communication | EA | Indirect - Maternal | -0.01967 | -0.00393 | -0.0354 | 0.008027 | -2.45019 | 0.07142579 | FALSE |
| Social & communication | Cog. Ability | Direct | 0.007211 | 0.024324 | -0.0099 | 0.008731 | 0.825966 | 0.60122299 | FALSE |
| Social & communication | Cog. Ability | Indirect - Paternal | -0.017 | -0.00214 | -0.03185 | 0.00758 | -2.24244 | 0.07199135 | FALSE |
| Social & communication | Cog. Ability | Indirect - Maternal | -0.00704 | 0.008311 | -0.02239 | 0.007832 | -0.89887 | 0.54122032 | FALSE |
| Social & communication | Dyslexia | Direct | -0.00289 | 0.014042 | -0.01982 | 0.008638 | -0.33428 | 0.88010333 | FALSE |
| Social & communication | Dyslexia | Indirect - Maternal | -0.00488 | 0.010243 | -0.02001 | 0.007717 | -0.63276 | 0.67551216 | FALSE |
| Social & communication | Dyslexia | Indirect - Paternal | 0.016403 | 0.031328 | 0.001477 | 0.007615 | 2.153989 | 0.08223866 | FALSE |

p.value.adj – adjusted p value correction (FDR); sig after MT – True/False value if statistically significant after multiple testing corrections

Supplementary Table 11: Variance explained for direct vs. indirect effect in multi-trait trio-PGS models using PGS calculated with preregistered PRScise2.

| Scale | % variance explained direct | % variance explained indirect | ratio direct:indirect | % variance explained direct no sibs | % variance explained indirect no sibs | ratio direct:indirect no sibs |
| --- | --- | --- | --- | --- | --- | --- |
| Social & communication (SCQ) | 0.021 | 0.097 | 0.22 | 0.023 | 0.094 | 0.24 |
| RRBI (SCQ) | 0.004 | 0.042 | 0.1 | 0.006 | 0.049 | 0.12 |
| Attention (CBCL) | 0.133 | 0.081 | 1.64 | 0.122 | 0.071 | 1.72 |
| Hyperactivity (CBCL) | 0.045 | 0.063 | 0.71 | 0.054 | 0.068 | 0.79 |
| Motor (ASQ) | 0.027 | 0.14 | 0.19 | 0.029 | 0.132 | 0.22 |
| Language (ASQ) | 0.087 | 0.085 | 1.02 | 0.095 | 0.098 | 0.97 |

No sibs – models only include one sibling per family

# Supplementary References

Choi, S. W., & O’Reilly, P. F. (2019). PRSice-2: Polygenic Risk Score software for biobank-scale data. *GigaScience*, *8*(7), giz082. https://doi.org/10.1093/gigascience/giz082

Coombes, B. J., Ploner, A., Bergen, S. E., & Biernacka, J. M. (2020). A principal component approach to improve association testing with polygenic risk scores. *Genetic Epidemiology*, *44*(7), 676–686. https://doi.org/10.1002/gepi.22339

Hegemann, L., Corfield, E. C., Askelund, A. D., Allegrini, A. G., Askeland, R. B., Ronald, A., Ask, H., St Pourcain, B., Andreassen, O. A., Hannigan, L. J., & Havdahl, A. (2024). Genetic and phenotypic heterogeneity in early neurodevelopmental traits in the Norwegian Mother, Father and Child Cohort Study. *Molecular Autism*, *15*(1), 25. https://doi.org/10.1186/s13229-024-00599-0

Privé, F., Albiñana, C., Arbel, J., Pasaniuc, B., & Vilhjálmsson, B. J. (2023). Inferring disease architecture and predictive ability with LDpred2-auto. *The American Journal of Human Genetics*, *110*(12), 2042–2055. https://doi.org/10.1016/j.ajhg.2023.10.010

Privé, F., Arbel, J., Aschard, H., & Vilhjálmsson, B. J. (2022). Identifying and correcting for misspecifications in GWAS summary statistics and polygenic scores. *Human Genetics and Genomics Advances*, *3*(4). https://doi.org/10.1016/j.xhgg.2022.100136

Privé, F., Arbel, J., & Vilhjálmsson, B. J. (2021). LDpred2: Better, faster, stronger. *Bioinformatics*, *36*(22–23), 5424–5431. https://doi.org/10.1093/bioinformatics/btaa1029
